# Dual species sphingosine-1-phosphate lyase inhibitors to combine antifungal and anti-inflammatory activities in cystic fibrosis: a feasibility study

**DOI:** 10.1038/s41598-023-50121-4

**Published:** 2023-12-20

**Authors:** Barbara Cellini, Gioena Pampalone, Emidio Camaioni, Marilena Pariano, Flavia Catalano, Teresa Zelante, Mirco Dindo, Lara Macchioni, Alessandra Di Veroli, Roberta Galarini, Fabiola Paoletti, Magdalena Davidescu, Claudia Stincardini, Gianluca Vascelli, Marina Maria Bellet, Julie Saba, Stefano Giovagnoli, Giorgio Giardina, Luigina Romani, Claudio Costantini

**Affiliations:** 1 Department of Medicine and Surgery, University of Perugia, P.le Lucio Severi 1, 06132 Perugia, Italy; 2 Department of Pharmaceutical Sciences, University of Perugia, Perugia, Italy; 3 Department of Biochemical Sciences “A. Rossi Fanelli”, Sapienza University of Rome, Rome, Italy; 4 Department of Chemistry, Biology and Biotechnology, University of Perugia, Perugia, Italy; 5 Centro Sviluppo e Validazione Metodi, Istituto Zooprofilattico Sperimentale dell’Umbria e delle Marche “Togo Rosati”, Perugia, Italy; 6 Department of Pediatrics, University of California San Francisco, San Francisco, CA USA

**Keywords:** Biochemistry, Fungal host response, Cystic fibrosis, Drug development

## Abstract

Cystic fibrosis (CF) is an autosomal recessive disorder characterized by respiratory failure due to a vicious cycle of defective Cystic Fibrosis Transmembrane conductance Regulator (CFTR) function, chronic inflammation and recurrent bacterial and fungal infections. Although the recent introduction of CFTR correctors/potentiators has revolutionized the clinical management of CF patients, resurgence of inflammation and persistence of pathogens still posit a major concern and should be targeted contextually. On the background of a network-based selectivity that allows to target the same enzyme in the host and microbes with different outcomes, we focused on sphingosine-1-phosphate (S1P) lyase (SPL) of the sphingolipid metabolism as a potential candidate to uniquely induce anti-inflammatory and antifungal activities in CF. As a feasibility study, herein we show that interfering with S1P metabolism improved the immune response in a murine model of CF with aspergillosis while preventing germination of *Aspergillus fumigatus* conidia. In addition, in an early drug discovery process, we purified human and *A. fumigatus* SPL, characterized their biochemical and structural properties, and performed an in silico screening to identify potential dual species SPL inhibitors. We identified two hits behaving as competitive inhibitors of pathogen and host SPL, thus paving the way for hit-to-lead and translational studies for the development of drug candidates capable of restraining fungal growth and increasing antifungal resistance.

## Introduction

Cystic fibrosis (CF) is an autosomal recessive disorder caused by mutations in the gene encoding the Cystic Fibrosis Transmembrane conductance Regulator (CFTR), a chloride/bicarbonate channel that regulates the electrolyte content of luminal fluid. Mutations in CFTR result in viscid secretion and defective airway mucociliary clearance, thus providing a suitable environment for persistent microbial colonization^1^. The chronic inflammatory state of the lungs in CF patients and the defective immune response further promote microbial infection in a vicious cycle that perpetuates the decline of the respiratory functions^2^. It is becoming increasingly clear that the development of anti-microbial therapies should be paralleled by the identification of drugs that target the defective inflammatory response^2^. Considering the high treatment burden of CF patients^3^, it would be ideal to identify drugs with multiple activities, able to curb microbial pathogenicity while potentiating the immune defence.

Pulmonary exacerbations are usually caused by bacteria typically associated with CF, although the prevalence of other pathogens such as fungi has increased over the past decades^4^, with *Aspergillus fumigatus* being by far the most common fungal species isolated in the sputum of CF patients^4,5^. There are currently four classes of antifungals for the treatment of systemic infection, and they all target fungi-specific components^6^. However, novel agents are being developed that target common pathways in the host and the pathogen. In this case, the selective inhibition of the fungal enzyme over the human counterpart is essential for effective antifungal drug development^6^. The selectivity might be achieved by resorting to the structural differences of the target between the host and the pathogen or to the specific context in which the target operates, i.e. a network-based drug selectivity^7^. In the latter case, it is possible to target a metabolic pathway common to the host and the pathogen, and take advantage of the specific network to harm the pathogen while leaving the host unaffected^7^, or, even more desirable, providing benefits to the host, such as by increasing its defence mechanisms.

Sphingolipids are ubiquitous components of eukaryotic cell membranes, and their metabolites play a role as signalling molecules^8^. The de novo biosynthetic pathway is initiated by the enzyme serine palmitoyltransferase and leads to the formation of ceramide (Cer), a central signalling molecule. Deacylation of Cer to sphingosine and subsequent phosphorylation by sphingosine kinase lead to the formation of sphingosine-1-phosphate (S1P), whose irreversible degradation by S1P lyase (SPL) terminates sphingolipid metabolism^8^. Accumulating evidence indicates that the Cer/S1P rheostat is important for lung development and physiology by regulating cell survival, vascular barrier function, and proper host response to airway microbial infections^9^, and S1P has emerged as a novel target for lung disorders^10^. In the context of CF, multiple defects in sphingolipid metabolism have been identified that might play a role in the pulmonary manifestations of the disease, in particular in lung inflammation and susceptibility to infection^11,12^, including reduced levels of S1P^13,14^. Indeed, in a murine model of CF, the administration of an SPL inhibitor could restore S1P levels and promote the resolution of inflammation upon LPS-fMLP challenge^13^. Sphingolipids are also essential constituents of fungi with a variety of functions, including fungal pathogenesis, and represent potential new targets for the development of antifungal drugs^15^. Among others, SPL represents an interesting target, because its inhibition would increase fungal S1P, which is highly toxic to fungi^15–17^.

SPL (EC 4.1.2.27) is a pyridoxal 5′-phosphate (PLP)-dependent enzyme bound to the endoplasmic reticulum membrane through an N-terminal sequence, with the catalytic domain facing the cytosol^18^. The protein is a tight dimer and belongs to the Fold Type I family. PLP is bound through a Schiff base linkage with a lysine residue that in the human enzyme complexed with a known inhibitor is Lys359 (internal aldimine)^19,20^. The active site is formed by residues from both subunits and comprises an Y-shaped channel whose distal part is largely hydrophobic^20^. The cofactor binding mode and the active site in general are very well conserved between human and yeast SPL^19^. Based on these premises, we have investigated whether dual-species SPL inhibitors, able to target both *A. fumigatus* SPL (AfuSPL) and human SPL (hSPL), could be identified that would result in a more potent antifungal activity by enhancing the immune response of the host while reducing the fitness of the pathogen. Herein, we show in vivo and in vitro experiments supporting S1P modulation as suitable strategy to counteract inflammation and infection in CF, and we characterized the biochemical and structural properties of AfuSPL and hSPL in the recombinant purified form. From 50 putative compounds, selected through a virtual screening campaign, we identified two competitive inhibitors for both hSPL and AfuSPL with K_I_ in the high micromolar range.

## Materials and methods

### Materials

The pET28 vector encoding Δ1-81AfuSPL was purchased from Twin Helix. *E.coli* pGro7 cells transformed with pET28 vector encoding Δ1-81 hSPL were kindly obtained from Prof. Riccardo Percudani of the University of Parma. Arabinose, Lactose, Glucose, PLP, Betaine, 1,4-dithiothreitol (DTT), Kanamycin, Chloramphenicol, Imidazole, NaCl, Sucrose, Adenosine 5′-triphosphate (ATP), NaOH, TRIZMA, HEPES, Triton X-100, EDTA, Potassium phosphate, Glycine, Glycerol, Methanol, KCl, Albumine from bovine serum (BSA) and mPAGE^®^ Unstained Protein Standard were purchased from Sigma-Aldrich. ANS (8-anilino-1-naphthalenesulfonic acid) was purchased from Molecular Probes. EDTA-free protease Inhibitor Cocktail was purchased from Roche. The S1P fluorogenic substrate was purchased from Cayman Chemical (CAS Number: 1166838-84-1). MgCl_2_ was purchased from Fluka. Luria Broth was purchased from Condalab. Anti-His (C-term)-HRP antibody Novex was purchased from Life Technologies. All chemicals were of the highest purity available.

### Mice, infection and treatments

All experimental protocols were approved by the Organism for the animal wellbeing (OPBA) of the University of Perugia, Italy, and the Italian Ministry of Health (Authorization number 1017/2020-PR). All methods are reported in accordance with ARRIVE guidelines (https://arriveguidelines.org). CF mice homozygous for the Phe508del-Cftr allele, were obtained from B. Scholte (Erasmus Medical Center, Rotterdam, Netherlands) and generated as previously described^21^. Mice were housed in a controlled environment at the Animal Facility of the University of Perugia. 6–8 week-old male and female mice were used for the experiments. For *A. fumigatus* infection, mice were anesthetized in a plastic cage by inhalation of 3% isoflurane (Forane, Abbott srl, Rome, Italy) in oxygen before intranasal instillation of 2 × 10^7^ *Aspergillus fumigatus* (Af293) resting conidia per 20 μl of saline. Naïve mice were used as control group. Quantification of fungal growth was done as described^22^. Bronchoalveolar lavage (BAL) fluid was collected in a plastic tube on ice and centrifuged at 4 °C for 5 min. For differential BAL fluid cell counts, cytospin preparations were made and stained with May-Grünwald Giemsa reagents (Sigma-Aldrich). For histology, paraffin-embedded sections were stained with periodic acid–Schiff (PAS). Mice were treated intranasally with SiRNA against murine SPL before challenging them with *A. fumigatus* conidia, and nonspecific SiRNA duplex were used as controls.

### Ethical approval

This study was carried out in compliance with the ARRIVE guidelines and all methods were carried out in accordance with relevant guidelines and regulations.

### SiRNA design and delivery

Predesigned SiRNA against *Sgpl1* (mm.Ri.Sgpl1.13.1) was purchased from Integrated DNA Technologies (IDT) (TEMA Ricerca, Italy). For in vivo studies, each mouse was lightly anesthetized by inhaled diethyl ether, then given an intranasal administration of unmodified SiRNA (10 µg/kg) or equivalent doses of nonspecific control SiRNA duplex in a volume of 20 μL of duplex buffer (IDT, TEMA ricerca). Intranasal SiRNA was given one day before infection and 2 and 4 days after infection.

### Lipid extraction and data analysis

Lyophilized lung tissues were kept frozen at −80 °C until analysis. An aliquot was thawed on ice and weighed before transferring to a clean Eppendorf tube for the extraction. Extraction for untargeted lipidomic analysis was performed as described in^23^. Briefly, a specific volume (2 ml for 0.2 g of tissue) of the mixture of organic solvents MMC (Methanol:MTBE:Chloroform, 40:30:30, v:v:v), containing BHT (100 mg/l) as antioxidant and the Standard mixture (2.5 µg/ml) Equisplash Lipidomix (Avanti Polar Lipids) was added to each samples. Samples were then vortexed and incubated at 950 rpm for 30 min at RT in a Thermomixer. Samples were centrifuged for 10 min, RT, 8000 rpm and supernatants were collected. An aliquot of 2 µl was injected into an LC–MS/MS system for analysis.

The LC/MS system consisted of a Dionex UltiMate 3000 series (Thermo Fisher Scientific, Waltham, MA, USA) with a binary pump, a thermostated autosampler and a column compartment coupled with a Thermo Q-exactive mass spectrometer (Thermo Fisher Scientific, Waltham, MA, USA). Liquid chromatography separation was performed at 45 °C using a Kinetex F5 reverse-phase column (Phenomenex Inc., Bologna, Italy) at the flow rate of 0.65 ml/min. The mobile phases consisted of 5 mM ammonium formate and 0.1% formic acid in water (solvent A) and in isopropanol (solvent B) with the following gradient: 0 min, B 20%; 3 min, B 40%; 16 min, B 60%; 16.5 min, B 70%; 24 min, B 74%; 28 min, B 95%; and 30 min, stop run. All solvents were purchased from Sigma-Aldrich.

Mass spectrometry analysis was performed as a first step in the positive/negative ion switching mode in full MS scan mode. Then, the raw data were processed with Lipostar^24^ software (version 2.1.1, Molecular Discovery Ltd., UK) to perform a pre-identification (by mass searching within a library of approximately 800,000 in silico fragmented lipids) of potential lipid species considering the mass and retention time values. The masses of interest were used for an inclusion list used in DDA mode with a reduced number of samples (automatically selected by Lipostar) to obtain MS/MS data. These were then imported into the data matrix generated by Lipostar to perform the final lipid identification step. Automatically generated data were visually inspected, and only high-confidence data were kept in the matrix and used for the statistical analysis.

Principal Component Analysis (PCA) was also performed using the Lipostar software (Molecular Discovery, UK, v 1.3.4), and Pareto scaling was applied.

### Fungal germination

Fungal strain *A. fumigatus* (Af293) was grown on potato dextrose agar for 5 days at RT, collected with a cell scraper and resuspended in PBS/tween 20 (0.05%). Germination was assessed in liquid Iscove's Modified Dulbecco's Medium (IMDM) and IMDM supplemented with d-*erythro*-sphingosine (0.1 and 1 mM). Briefly, 1 × 10^5^ spores/mL in IMDM and IMDM supplemented with d-*erythro*-sphingosine were inoculated into each well of a 24-well dish overnight at 28 °C. Microscopic images were captured using an Evos inverted microscope. Germinated spores were observed using a 40× objective and phase-contrast images. Values in figures represent the average percentage of spores germinated ± SD.

### Protein expression and purification

*E. coli* BL21 cells were co-transformed with a pGro7 plasmid encoding GroEL and GrosES chaperones and the constructs pET28 Δ1-80 hSPL and pET28 Δ1-81 AfuSPL encoding the cytosolic catalytic domain of human SPL and *A. fumigatus* SPL, respectively. 2 l of Luria broth, supplemented with arabinose 1 g/l, lactose 2 g/l, glucose 0.5 g/l, PLP 100 μM, betaine 10 mM and additioned with kanamicin 35 mg/l and chloramphenicol 68 mg/l antibiotics, were grown at 37 °C to an absorbance at 600 nm of 0.4–0.6. Protein expression was induced by decreasing the temperature to 20 °C for 15 h. Cells were harvested by centrifugation at 4300*g* for 10 min at 4 °C and resuspended in Tris–HCl 50 mM pH 8.0, NaCl 500 mM, imidazole 40 mM, PLP 100 μM, 2× EDTA-free protease inhibitors (Roche). The homogenate was then lysed by ten cycles of sonication for 30 s on ice at 18 microns amplitude, and the suspension was centrifuged at 18,000*g* for 30 min at 4 °C. The soluble fraction was filtered and loaded on a HisPrep 16/10 affinity chromatography column (Cytiva) pre-equilibrated with the loading buffer Tris–HCl 50 mM pH 8.0, NaCl 500 mM, imidazole 40 mM. Subsequently, a column wash was carried out with five column volumes of Tris–HCl 50 mM pH 8.0, KCl 100 mM, sucrose 500 mM, MgCl_2_ 20 mM and ATP 5 mM to remove chaperones co-expressed with the protein of interest. Upon re-equilibration with the loading buffer, a linear gradient was applied from 0 to 100% in 100 mL of Tris–HCl 50 mM pH 8.0, NaCl 500 mM, 500 mM imidazole. Soluble SPL eluted between 100 and 150 mM imidazole as a single symmetrical peak. Fractions containing the protein were pooled, reconstituted with 300 μM PLP, and concentrated using Vivaspin Turbo 15 devices (Sartorius). A second step of purification was performed using a HiPrep 16/60 Sephacryl S-300 HR size-exclusion chromatography column pre-equilibrated in Tris–HCl 20 mM pH 8.0, NaCl 150 mM, DTT 1 mM, glycerol 5%. Eluted SPL was concentrated using Vivaspin Turbo 15 devices and stored at −80 °C where it is stable for at least three months.

SDS-PAGE and western-blot analyses were performed to evaluate the purity of the protein. 10 μg of protein were run on a 10% gel, stained with Coomassie dye and the images acquired using an iBright FL1500 Imaging System (Thermo Fisher Scientific). For western-blot, 0.25 μg of the purified proteins were loaded on a 10% SDS–PAGE and transferred on a nitrocellulose membrane. Gel loading was checked by Ponceau staining. The membrane was blotted with Anti-His (C-term)-HRP antibody (1:5.000) in 5% (w/v) BSA in TTBS (50 mM Tris–HCl pH 7.5, 150 mM NaCl, 0.1% Tween 20) overnight at 4 °C. After three washes of 15 min each in TTBS, immunocomplexes were quantified by an enhanced chemiluminescence (ECL, Pierce Biotechnology, Rockford, IL) using an iBright FL1500 Imaging System.

### Spectroscopic measurements

Absorbance spectra were registered using a Jasco V-750 spectrophotometer using 1 cm path length quartz cuvettes at a protein concentration of 5 μM in Tris–HCl 20 mM pH 8.0, NaCl 150 mM, DTT 1 mM, glycerol 5%. AfuSPL and hSPL concentrations in the holo-form were determined from absorbance at 280 nm using the molar absorption coefficient of 109,640 and 112,370 M^−1^ cm^−1^, respectively. PLP content was measured after treatment of the holoenzyme with 0.1 M NaOH using the molar absorption coefficient of 6600 M^−1^ cm^−1^ at 388 nm of free PLP. Fluorescence data were obtained using a 1 cm optical quartz cuvette in a Jasco FP-8200 fluorimeter with excitation and emission bandwidths of 5 nm. Fluorescence spectra of 1 µM SPL in the presence of 20 µM PLP, incubated with 20 mM ANS for 1 h at 25 °C, were registered upon excitation at 365 nm. All measurements were performed in Tris–HCl 20 mM pH 8.0, NaCl 150 mM, DTT 1 mM, glycerol 5% at 25 °C. Circular dichroism (CD) spectra were obtained using a Jasco J-810 spectropolarimeter with a thermostatically controlled cell compartment at 25 °C. For measurememts in the near-UV and visible wavelengths, protein concentration was 5 μM in the presence of 200 μM PLP in a cuvette with a path length of 1 cm. For measurements in the Far-UV region, protein concentration was 0.1 mg/ml in a cuvette with a path length of 0.1 cm.

### Lyase activity assay

The fluorimetric method used to measure SPL lyase activity was based on the dosage of umbelliferone produced during the reaction with a synthetic SPL fluorogenic substrate, as described previously^25^. 3 μg/ml AfuSPL or hSPL in 100 mM Hepes buffer, pH 7.4, containing 0.1 mM EDTA, 0.05% Triton X-100 and PLP 100 μM, were mixed with 5 mM S1P fluorogenic substrate, resuspended in 0.5 M potassium phosphate buffer, pH 7.2 (final volume: 10 μl). The mixture was then incubated at 37 °C for 1 h for the fungal enzyme and 2 h for the human enzyme, and the enzymatic reaction was stopped by the addition of 6 μl of MeOH. Then, 10 μl of assay mixture were diluted with 80 μl of assay buffer (100 mM Hepes buffer, pH 7.4, 0.1 mM EDTA, 0.05% Triton X-100) on 96-well plates (Greiner). 10 μl of 800 mM glycine–NaOH buffer, pH 10.6, were added to the resulting solution for fluorescence acquisition (final volume: 100 μl). The assay mixture was placed on a 96-well plate and incubated for 20 min at 37 °C. The resulting fluorescence signal was measured using a TECAN microplate reader (λ_ex_/_em_ = 360/450 nm), and the amount of umbelliferone formed was calculated using a calibration curve. The kinetic parameters of the lyase reaction were determined by measuring the activity at increasing substrate concentration (from 0.1 to 5 mM) using the procedure described above. Data reported as pmoles umbelliferone/μg enzyme/min were fitted to a Michaelis–Menten equation.

#### Inhibition studies

The 50 compounds identified by virtual screening were purchased from SPECS and dissolved in DMSO at 100 mM concentration. Inhibition studies were performed by measuring the residual lyase activity of both hSPL and AfuSPL in the presence of 2 mM substrate and each inhibitor at 1 mM concentration. In detail, the inhibition assay mixture contained 3 μg/ml AfuSPL or hSPL in 100 mM Hepes buffer, pH 7.4, containing 0.1 mM EDTA, 0.05% Triton X-100 and PLP 100 μM, 2 mM S1P fluorogenic substrate, 1 mM inhibitor (final volume: 10 μl). The assay was performed as described in the lyase activity assay section. Negative controls indicate vehicle-only conditions. The K_I_ value of dual inhibitors was calculated by measuring the enzyme kinetic parameters in presence of various inhibitor concentrations (0–1 mM). Obtained data were analyzed through a global fitting to a competitive inhibition model by using Prism 6.0 software (GraphPad software, San Diego, CA, USA).

### Homology model construction and validation

The primary sequence of AfuSPL was obtained from the National Center for Biotechnology Information (NCBI) database in FASTA format. We first built the AfuSPL structural model using the SWISS-MODEL portal (https://swissmodel.expasy.org)^26^, based on a target-template alignment. Upon searching the template library using BLAST^27^ and HHBlits^28^, the crystal structure of SPL from *Burkholderia pseudomallei* (PDB accession: 5K1R) was chosen as template. The global quality of the modeled structure was evaluated based on the rootmean-square deviation (RMSD) value, obtained by overlapping the peptide chain of the conserved regions of the generated model using UCSF Chimera^29^. The validation of the stereochemical quality of the model was determined by both the Ramachandran plot, which was generated using PROCHECK^30^, and scores provided by ERRAT^31^ and Verify3D^32^. Additional structural evaluations and stereo chemical analyses were performed using the ProSA-web server (https://prosa.services.came.sbg.ac.at/prosa.php^26^ and QMEAN^33^) on the SWISSMODEL portal. These tools correlate the scores obtained for various parameters of the constructed model, including local geometry, distance-dependent interaction potential, and solvation potential, with the corresponding scores of reference structures experimentally determined by X-ray crystallography.

### Virtual screening and molecular docking studies

All molecular modeling calculations were performed on Ubuntu 20.04 operating system running on an Intel-based workstation (i9, 20CPUs). The available structure of hSPL in complex with the inhibitor 30J (6-[(2*R*)-4-(4-benzyl-7-chlorophthalazin-1-yl)-2-methylpiperazin-1-yl]pyridine-3-carbonitrile)^20^ was instrumental for the molecular docking studies of the virtual screening (VS) campaign. To this end, an optimized VS protocol on the available crystallized hSPL, at the time we started the project, was developed as follows.

#### Protein preparation

hSPL file 4Q6R.pdb (resolution: 2.40 Å)^20^ was retrieved from the pdb database (https://www.rcsb.org) directly by the MolProbity server (http://molprobity.biochem.duke.edu) where the file was checked, hydrogen atoms added and the result was saved in the working folder as a new pdb file. Open Babel (ver. 3.0.0), a chemistry file translation program^34^, was then used to convert the pdb in the corresponding pdbqt and mol2 type files adding Gasteiger partial charges^35^.

#### Ligands preparation

3D coordinates of commercially available compounds (about 210 K molecules) were retrieved from the SPECS database (https://www.specs.net, accession July 2021) and converted in the corresponding pdbqt and mol2 type files by means of the Open Babel^34^ adding hydrogen atoms and Gasteiger charges.

#### Virtual screening and docking procedures

All prepared files were then passed to the molecular docking studies by using three softwares: AutoDockVina^36^, idock^37^, and PLANTS^38^. These docking programs were selected since they work with different searching engines and scoring functions (Monte Carlo algorithm, random forest, ant colony optimization, respectively), thus, consensus docking methodology was applicable^39^. Since hSPL is a homodimer (chains A and B) with two symmetrical catalytic sites, only one binding site, centered at the following cartesian coordinates (x: 35.7, y: 10.8, z: 22.6), was used in the docking simulations. A large searching space of interest was defined as a cubic box of 22 Å per side or, in the case of PLANTS, as a sphere with a radius of 12 Å. Other parameters used by the docking softwares were as default. As results, the first 100 virtual hits (vHits) showing high scores (lower binding energy) comparable from all three docking methods were selected. Finally, visual inspection by using USCF-Chimera (ver. 1.15)^29^ of the docking poses, similarity search over the best vHits discovered in the preliminary assays, and the availability from the vendor led to the acquisition of 50 compounds that were further studied and tested on biochemical assays.

The discovered hit C17 was then redocked on the new obtained crystals (hSPL-8AYF, AfuSPL-8CMX) by using AutoDockVina as described above. Briefly, the 3D coordinates of both proteins were checked, corrected and hydrogen atoms added by means of MolProbity server (http://molprobity.biochem.duke.edu) and then the obtained pdb files were converted to pdbqt format as above reported. AutoDockVina^36^ was used as docking software by using the parameters reported in Table S1. Finally, binding poses, with the lowest docked energy, and the resulted putative complexes were analyzed and rendered with LigPlot+ (ver. 2.2.8)^40^ and USCF-Chimera^29^. All molecular modeling files are available from the authors upon request.

### Crystallization, data collection, phasing, model building and refinement

Crystals of hSPL and AfuSPL were obtained by vapour diffusion methods from Morpheus screen (Molecular Dimensions, Rotherham, UK) and from Crystal Screen (Hampton Research, Aliso Viejo, CA, USA), respectively: crystallization conditions are reported in Table S2. X-ray diffraction data were collected at Elettra synchrotron (Trieste, IT) on beamline XRD2. For both proteins data were processed using the automated data processing pipeline available at the synchrotron, comprising the following software: autoPROC v 1.0.5^41^, XDS^42^, POINTLESS v.1.12.12^43^, CCP4 suite v.7.1.018^44^. The data were scaled anisotropically using STARANISO v.2.3.87^45^ to a maximum resolution of 1.84 Å for hSPL and 3.46 Å for AfuSPL. For hSPL, initial phases were obtained by molecular replacement (MR) using PDB 4q6r as template in Molrep v.11.9.02^46^, whereas for AfuSPL, the high-resolution structure of hSPL (this work) was used as template. Cycles of model building and refinement were performed with Coot v.0.9.8.1^47^ and Refmac v.5.8.0403^48^. The N-terminus region of AfuSPL was modelled into a clear positive electron density starting from an AlfaFold model generated using the ColabFold server^49,50^. For AfuSPL, 10% of reflection instead of default 5% were used to calculate R_free_, and additional geometric restraints were introduced during refinement using ProSMART v.0.86^51^, due to the low resolution of the diffraction data. Both structures were validated and deposited in the protein data bank with the following accession codes: hSPL-8AYF, AfuSPL-8CMX. Final statistics are reported in Table S2.

### Statistical analysis

Unpaired t-test and one-way analysis of variance with Dunnett’s post hoc test were used to determine the statistical significance. Significance was defined as p < 0.05. Data are pooled results or representative images. The in vivo groups consisted of seven to nine mice/group. Mice were allocated in each group by simple randomization. No criteria were set for including/excluding animals during the experiments. GraphPad Prism software V.6.01 (GraphPad Software) was used for analysis.

## Results

### *Aspergillus* infection alters the lipid profile in the lungs of a murine model of CF

**Figure 1:**
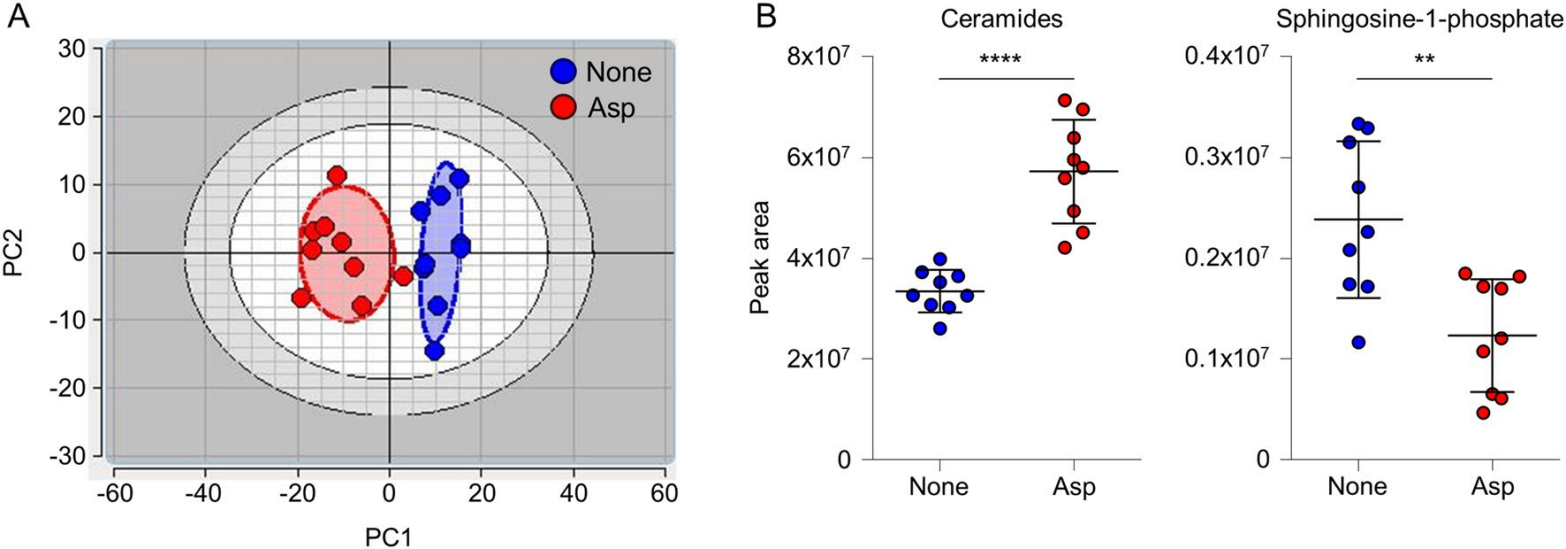
The Cer/S1P rheostat in the lungs of CF mice is perturbed upon *Aspergillus* infection. CF mice were infected intranasally with *A. fumigatus* and the lungs assessed at 7 days post-infection by untargeted lipidomics. (**A**) Score plot of the principal component analysis (PCA) carried out on a fingerprint of about 480 lipids. (**B**) Peak areas of ceramides (left panel) and sphingosine-1-phosphate (right panel) in untreated (None, blue dots) and *Aspergillus*-infected (Asp, red dots) mice. Data are expressed as mean ± SD. **p < 0.01; ****p < 0.0001, unpaired t test.

It has been reported that the levels of S1P are reduced in the lung of CF mice compared to wild-type littermates^13^. To extend these findings in the context of *Aspergillus* infection, CF mice were challenged or not with *Aspergillus* conidia for 7 days and the lungs analysed by untargeted lipidomics. As shown in Fig. 1A, the score plot of the Principal Component Analysis (PCA), carried out on a fingerprint of about 480 lipids, shows that the lungs of naïve and *Aspergillus*-treated mice clustered separately on the first component, indicating that the two lipid profiles are indeed distinct (PC1 explains 45.87% of the variance).

We then focused on the Cer/S1P rheostat. The levels of ceramides were significantly increased in the *Aspergillus*-treated group compared to naïve mice (Fig. 1B). Conversely, the levels of S1P were tenfold lower, and this lipid was sorted out of the final lipid fingerprint due to the stringent quality controls of the data processing. Nevertheless, upon examining the signal of this lipid from raw data, it is noteworthy that a significant reduction in the *Aspergillus*-treated group compared to naïve mice can be observed (Fig. 1B), thus supporting a perturbation of the Cer/S1P rheostat upon challenging with *Aspergillus* conidia.

All in all, these results indicate that *Aspergillus* infection changes the lipid profile in the lungs of CF mice, including a reduction in the levels of S1P, thus supporting the use of SPL inhibitors in CF.

### Host- and fungal-specific modulation of S1P levels supports the development of dual-species SPL inhibitors

**Figure 2:**
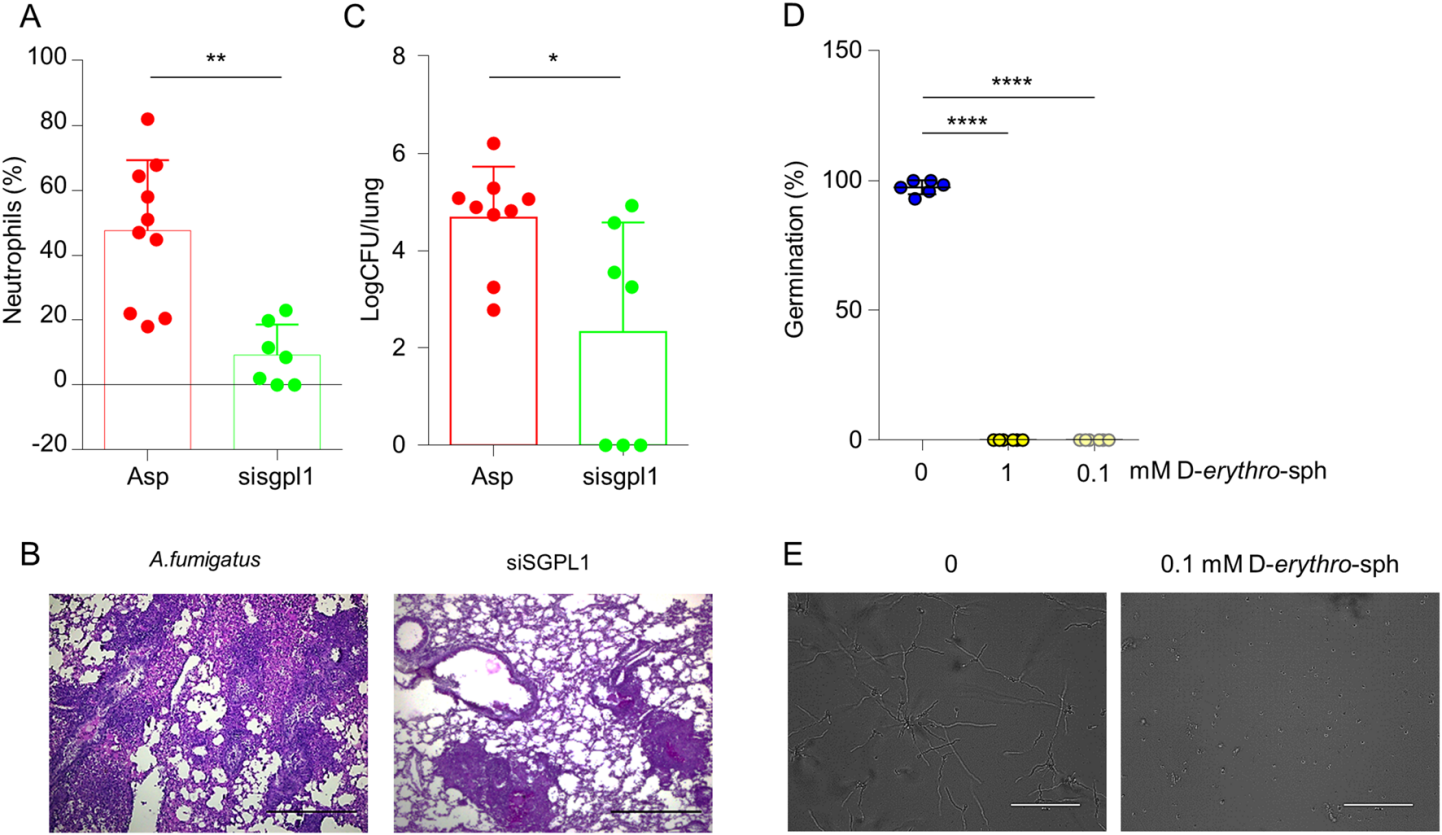
Anti-inflammatory and anti-fungal effects are achieved, respectively, by host and fungal-specific modulation of S1P metabolism. (**A-C**) CF mice were infected intranasally with live *A. fumigatus* conidia and treated with sisgpl1 in a volume of 20 µl of duplex buffer before evaluation of (**A**) neutrophils (%) in the bronchoalveolar lavage fluid, (**B**) lung histopathology at 7 dpi, and (**C**) fungal growth (log10 CFU mean ± SD). Scale bar, 500 µm. *, p < 0.05; **, p < 0.01, Mann–Whitney test. (**D,E**) Germination of *A. fumigatus* was assessed in liquid IMDM and IMDM supplemented with d-*erythro*-sphingosine (1 and 0.1 mM) using a ×40  objective and phase-contrast images captured after 20 h of culture. Values in (**D**) represent the average percentage of spores germinated ± SD. ****, p < 0.0001. Representative images are shown in (**E**).

The development of dual-species SPL inhibitors is based on the hypothesis that increasing S1P levels in the host and the fungus enhances the immune response in the former and reduces the fitness of the latter. To assess this hypothesis, we first treated mice intranasally with a SiRNA against murine SPL one day before challenging them with *A. fumigatus* conidia as well as at 2 and 4 days post-infection resulting in a 58% mean reduction in *sgpl1* mRNA levels (sisgpl1 vs Asp). As a result we found that administration of a SiRNA against murine SPL reduced neutrophil infiltration in the bronchoalveolar lavage (Fig. 2A) and improved histopathology (Fig. 2B) indicating a decreased inflammatory response. In this context, we also observed a variable, although statistically significant, reduction of the fungal burden (Fig. 2C). In this regard, it should be noted that the fungal burden becomes of secondary importance as the eventual persistence of fungi does not result in lung pathology.

We then cultured *Aspergillus* conidia in the presence or absence of d-*erythro*-sphingosine, which is likely converted to S1P as previously shown in *Saccharomyces cerevisiae*^52^. As shown in Fig. 2D, E, administration of d-*erythro*-sphingosine has a strong fungistatic effect, confirming the potential of S1P as an antifungal agent.

In conclusion, our results extend previous findings on the role of S1P in the regulation of the immune response in CF as well as on its toxic effects on fungal growth, thus supporting the development of dual-species SPL inhibitors as a combined anti-inflammatory and antifungal strategy in CF.

### Biochemical studies on hSPL and AfuSPL confirm that a dual targeting strategy is feasible

To validate the rationale of a dual-species SPL inhibition, we performed preliminary in silico analyses to compare the structural features of the two orthologous enzymes. The three-dimensional structure of human SPL in complex with the inhibitor 30J was already available (pdb id: 4Q6R)^20^. Using the SWISS-MODEL portal, we determined and validated the putative structure of AfuSPL by homology modelling. We chose as reference the solved structure of SPL from *Burkholderia pseudomallei* (pdb id: 5K1R) because of the high resolution (2.1 Å), the presence of bound PLP, and the good sequence identity with the fungal orthologue (44%). Our model (z-score −8.41, profile 3D score 87.63%, 99.2% of residues in a favoured position of the Ramachandran Plot) predicts that AfuSPL is dimeric, belongs to the Fold Type I family of PLP-dependent enzymes^53^, and shares the topology of the active site with hSPL (Fig. S1). The latter observations support the idea that a single molecule able to bind both enzymes could be identified, although subtle differences in the active site entrance might change binding specificity and affinity between the two orthologues.

**Figure 3:**
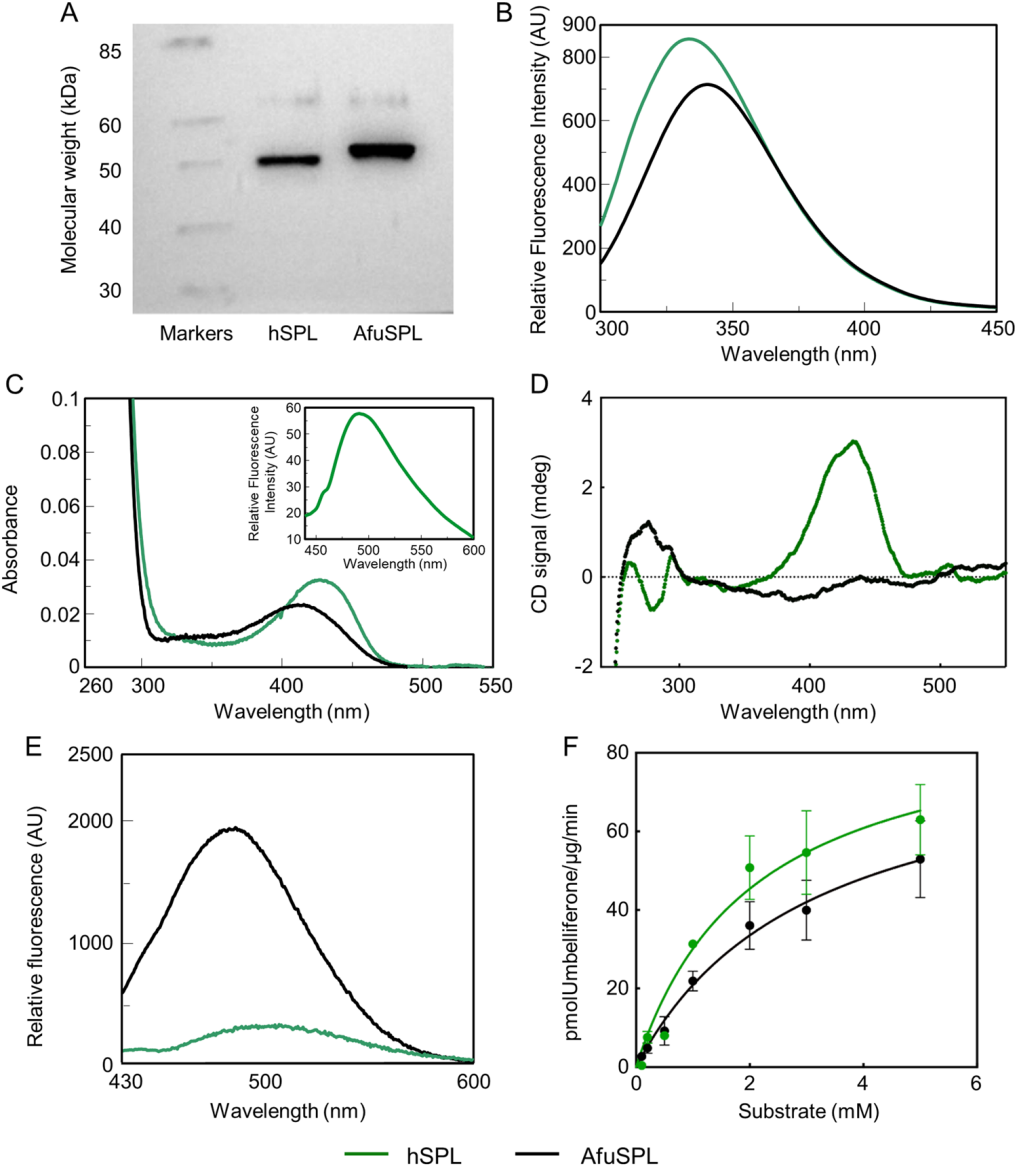
Western blot analyses, spectral features and kinetic properties of human and AfuSPL. (**A**) Western blot analyses of purified hSPL and AfuSPL. (**B**) Intrinsic emission fluorescence (excitation wavelenght at 280 nm) of 5 µM hSPL and AfuSPL. (**C**) Absorbance spectra of 5 μM hSPL (green line) and AfuSPL (black line). In the inset, internal aldimine emission fluorescence spectra (excitation wavelenght at 426 nm) of 5 μM hSPL. (**D**) Circular dichroism spectra of 5 μM hSPL and AfuSPL in the presence of 200 μM PLP. (**E**) Fluorescence emission spectra of 1 µM enzymes in the presence of 20 µM PLP incubated with 1 mM ANS at 25 °C for 1 h and registered upon excitation at 365 nm. All spectral measurements were made in 20 mM Tris–HCl pH 8.0, 150 mM NaCl, 1 mM DTT, 5% Glycerol. (**F**) Kinetic parameters for the lyase reaction of hSPL (green dots and line) and AfuSPL (black dots and line) in Hepes 100 mM buffer, pH 7.4, EDTA 0.1 mM, Triton X-100 0.05%. The graph shows the enzyme activity measured for hSPL and AfuSPL as a function of substrate concentrations (expressed as pmol umbelliferone/μg/min). The lines represent the fitting of the data to the Michaelis–Menten equation.

Therefore we expressed in *E. coli* and purified the catalytic domain of hSPL and AfuSPL endowed with a histidine tag at the C-terminus and characterized their biochemical properties. The resulting yield was 5 mg per liter of bacterial culture with 95% purity in the case of hSPL and 4 mg per liter of bacterial culture with 90% purity in the case of AfuSPL. Purified SPL migrates in SDS-PAGE as a single band with an apparent molecular mass of approximately 50 KDa, in agreement with the theoretical mass of the protein subunit, and responds to an antibody against the histidine tag (Fig. 3A and Fig. S2). We noticed that the yield of AfuSPL is lower as compared to hSPL, possibly due to a higher tendency to aggregation and PLP dissociation of AfuSPL (see below). Recombinant hSPL and AfuSPL bind 2 mol of PLP per dimer. Upon excitation at 280 nm, a characteristic emission maximum at 334 and 340 nm are observed for the human and the fungal enzymes, respectively, indicating a folded conformation (Fig. 3B). In the visible region, hSPL shows an absorbance maximum centered at 426 nm and a shoulder at about 340 nm, attributable to the ketoenamine and enolimine tautomers of the internal aldimine, respectively (Fig. 3C)^54^. The emission at 490 nm upon excitation at 426 nm confirms that the peak corresponds to a ketoenaminic PLP-Lys complex. Purified AfuSPL shows an absorbance maximum at 412 nm (inset of Fig. 3C). The shift of the visible absorbance band at lower wavelength as compared with hSPL can be attributed to a weaker interaction of the apoprotein with the coenzyme (Fig. 3C). Indeed, we found that the CD spectrum of hSPL shows a maximum at 430 nm, corresponding to the internal aldimine, and a negative band in the near-UV at 280 nm, reporting on aromatic amino acids in the proximity of the active site. On the other hand, AfuSPL shows a faint negative dichroic band around 400 nm, and displays a positive rather than negative dichroic band at 280 nm, a finding suggesting a different and possibly less rigid active site microenvironment as compared with the human counterpart (Fig. 3D). We also investigated the overall conformation of the two proteins by registering the fluorescence emission of the ANS probe, which specifically binds exposed hydrophobic patches^55^. As shown in Fig. 3E, the ANS fluorescence spectrum of hSPL shows low intensity with a maximum at ~500 nm, while that of AfuSPL shows a sixfold higher intensity as compared with hSPL and a shift of the maximum at ~ 490 nm, indicating the presence of hydrophobic regions on the fungal protein. Altogether, the absorbance, fluorescence and CD spectral data indicate that both hSPL and AfuSPL are folded, although the two active sites show subtle conformational differences, possibly resulting in a lower affinity for PLP in AfuSPL. Moreover, the two proteins are endowed with some differences in their physical–chemical properties, possibly related to the increased presence of hydrophobic surfaces on the fungal enzyme. Finally, by a fluorimetric activity assay based on a synthetic substrate, we determined the kinetic parameters for the lyase reaction catalyzed by hSPL (*k*_cat_ = 0.109 ± 0.014 s^−1^; K_m_ = 1.5 ± 0.4 mM) and AfuSPL (*k*_cat_ = 0.103 ± 0.007 s^−1^; K_m_ = 3.1 ± 0.4 mM) (Fig. 3F). The values are similar in the two proteins, thus further corroborating the similarity of their active sites. We also validated the assay by testing a known inhibitor of hSPL, 30J^20^, as a prototype of a molecule already optimized for the human enzyme^55^. At 1 mM concentration, it reduced the activity of hSPL by more than 85%, without any significant effect on the activity of AfuSPL (Fig. S3). This suggests that the accommodation of this molecule in the active site is different between the human and the fungal enzymes, supporting the search for new compounds as putative dual binding inhibitors.

### An integrated in silico prediction and wet-bench validation platform identifies dual-species SPL inhibitors

We resorted to in silico screening approaches for the identification of potential hits as dual-species SPL inhibitors. We performed high-throughput docking studies of hSPL with commercially available compounds (about 210,000 molecules) from the SPECS database (https://www.specs.net/). We applied a consensus docking methodology to help in improving the probability of identifying accurate docked poses^56^. This methodology is used with molecular docking in virtual screening campaigns to better filter potential ligands for a protein target. To this aim, the method exploits and combines results from different docking programs by averaging the rank of each virtual hit (vHit) obtained from each docking software. We retrieved 50 vHits (Table S3).

**Figure 4:**
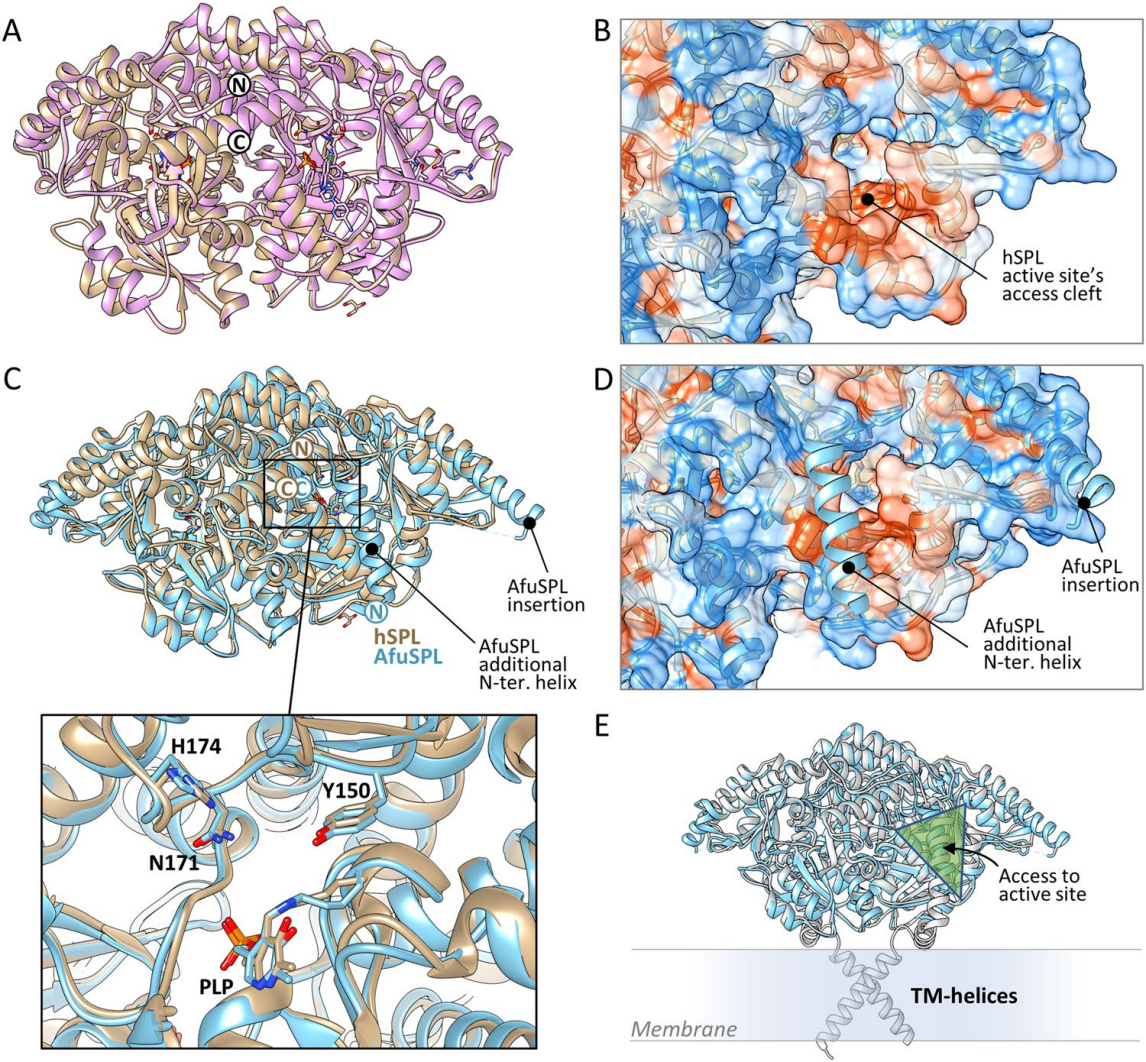
Crystal structures of unbound hSPL and AfuSPL. (**A**) Superposition of the inhibited (in pink; PDB 4Q6R^20^) and ligand free (in gold; this work) hSPL structures. The position of the C- and N-terminus are indicated. (**B**) Protein surface representation of hSPL entrance of the active site, coloured by amino acid hydrophobicity on the Kyte-Doolittle scale^75^ ranging from a maximum value of 4.5 for isoleucine (orange) to a minimum of −4.5 for arginine (blue). (**C**) Superposition of the solved hSPL (in gold) and AfuSPL (in blue). The structured N-terminal helix observed in AfuSPL but not in hSPL and the insertion of AfuSPL are highlighted. The position of the C- and N-terminus of the two structures are indicated with the same colours of the structures. A blow up of the PLP binding cleft with the residues that are likely involved in binding the phosphate group of S1P is shown below. (**D**) Position of the structured N-terminus helix and of the insertion of AfuSPL with respect to the substrate binding cleft. Protein surface is coloured by amino acid hydrophobicity as in (**B**). (**E**) Position of SPL with respect to the membrane. The scheme is based on the orientation of the N-terminus helix of AfuSPL. Given that AlfaFold predicts the presence of the same helix also for hSPL, it can be speculated that the trans-membrane regions (spanning from residue 41 to 61) must be on the same side of the protein.

To confirm the soundness of our virtual screening campaign, we solved the crystal structures of unbound hSPL and AfuSPL at 1.8 Å and 3.5 Å resolution, respectively. The structure of ligand-free hSPL was superposable with the one solved in complex with a known active site inhibitor with a RMSD of 0.36 Å (between 443 Cα pairs), suggesting that hSPL does not undergo extensive conformational changes upon ligand binding (Fig. 4A). Access to the active site is guaranteed by a deep cleft flanked by the last two C-terminal helices. The surface of this cleft is highly hydrophobic to accommodate the aliphatic tail of S1P (Fig. 4B). Therefore, the rigidity of the hSPL structure may account for the need to prevent a hydrophobic collapse that would close the substrate binding site. The structure of AfuSPL was solved at low resolution. Structural superposition with the human protein results in a RMSD of 0.82 Å (between 412 Cα pairs) (Fig. 4C and Fig. S4). Notably, the AfuSPL structure displays a structured N-terminal region, consisting of a long helix that is not observed in hSPL, although its presence in hSPL is predicted by AlfaFold^50^. This helix is positioned near the active site access cleft and, at least in the soluble construct, is much more flexible for the human protein compared to the fungal enzyme. Moreover, AfuSPL has an insertion of six residues in the C-terminal region that may also alter the dynamics of the substrate binding cleft (Fig. 4C, D and Fig. S4). On the other hand, no significant differences are observed at the active site level, where all the residues likely involved in substrate binding are conserved (Fig. 4C). Finally, it must be noted that the position of the additional N-terminal helix that is observed in the AfuSPL structure suggests that the protein binds to the membrane with an orientation that places the active site entrance on the opposite side with respect to the membrane surface (Fig. 4E).

Overall, the structural results confirm that, although some differences exist between human and fungal SPL, the S1P binding site is similar and may be targeted by the same compound.

Therefore, we tested the 50 candidates obtained from the virtual screening campaign for their inhibitory activity compared to the vehicle-only condition on both hSPL and AfuSPL in the recombinant purified form at a fixed concentration of 1 mM (Figure S5 and Table S4). Notably, we were not able to test compound C38 due to its high hydrophobicity that prevented the dissolution in the assay mixture. Moreover, compounds C12, C30 and C46 gave rise to fluorescence emission in the assay mixture, thus preventing a reliable measurement of umbelliferone production. The data obtained indicate that: (i) compounds C1-C3, C6-10, C13-C16, C18, C19, C22-27, C29, C31, C32, C34, C37, C39-C42, C44, C47-C50 only inhibit AfuSPL; (ii) compounds C4- C5, C11, C20-C21, C28, C35, C36, C43, C45, show a low inhibition potency on hSPL with a residual activity around 80%, and a more pronounced potency against AfuSPL with residual activity lower than 30%; (iii) compounds C17 and C33 show a statistically significant inhibitory activity on both proteins.

**Figure 5:**
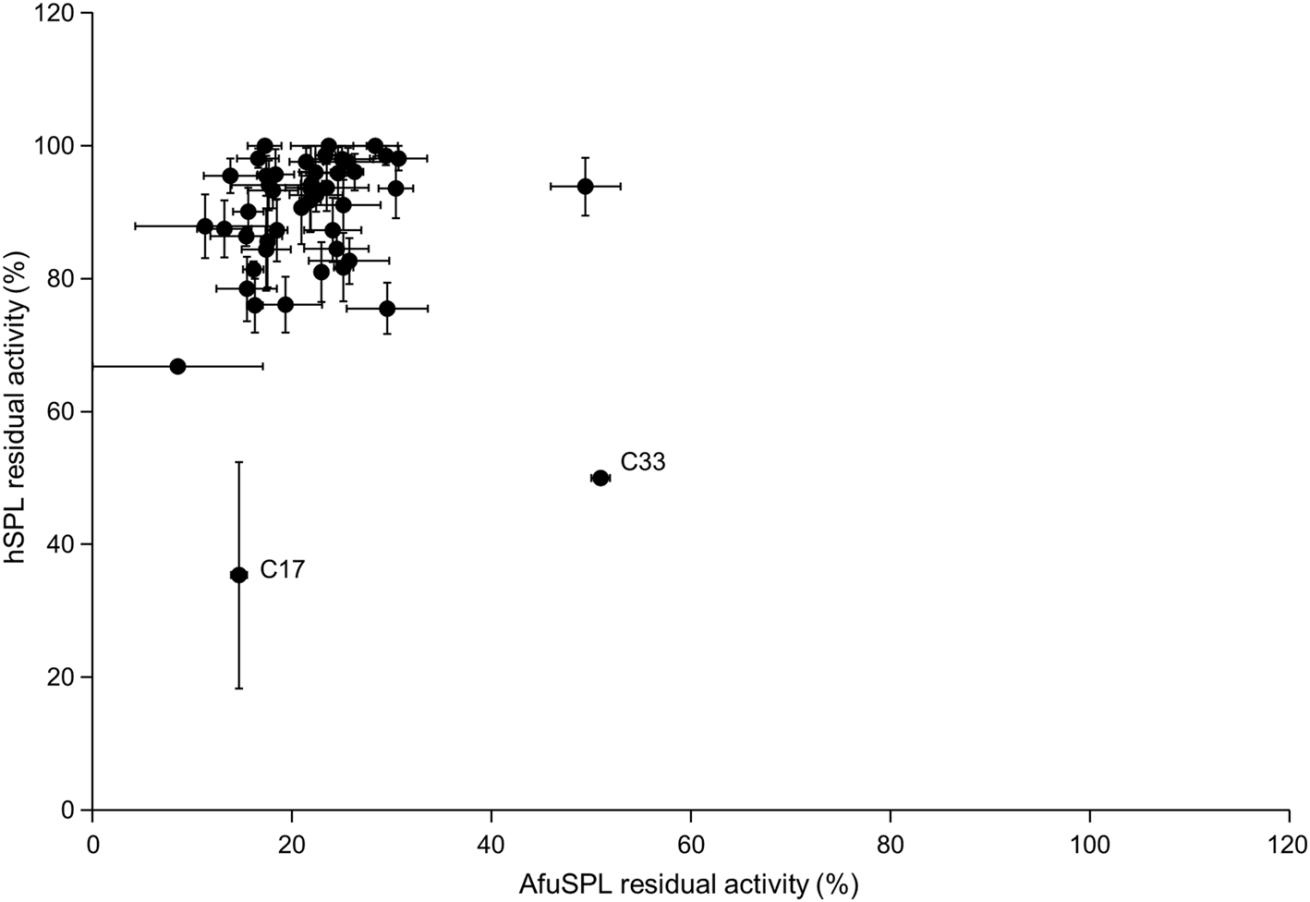
Compounds C17 and C33 display a dual-species inhibitory activity. Residual lyase activity of recombinant purified hSPL and AfuSPL was determined in the presence of each candidate selected by the virtual screening campaign at 1 mM concentration.

Upon ranking the compounds based on their inhibitory activity against hSPL and AfuSPL (Fig. 5 and Table S4), we focused our attention on compounds C17 and C33 (dual-target hit rate of 4%), whose main chemical features are shown in Table 1, because of their similar inhibition potency against the two targets.

**Table 1:** Chemical characterization of selected dual inhibitors.

Compounds	MW	cLogP	PSA (Å^2^)
	602.8	7.11	167.3
	553.0	6.4	106.6

The two hit compounds C17 and C33, purchased from the SPECS vendor, were analyzed by ^1^H-NMR spectroscopy to confirm high purity and structural identity (Fig. S6).

**Figure 6:**
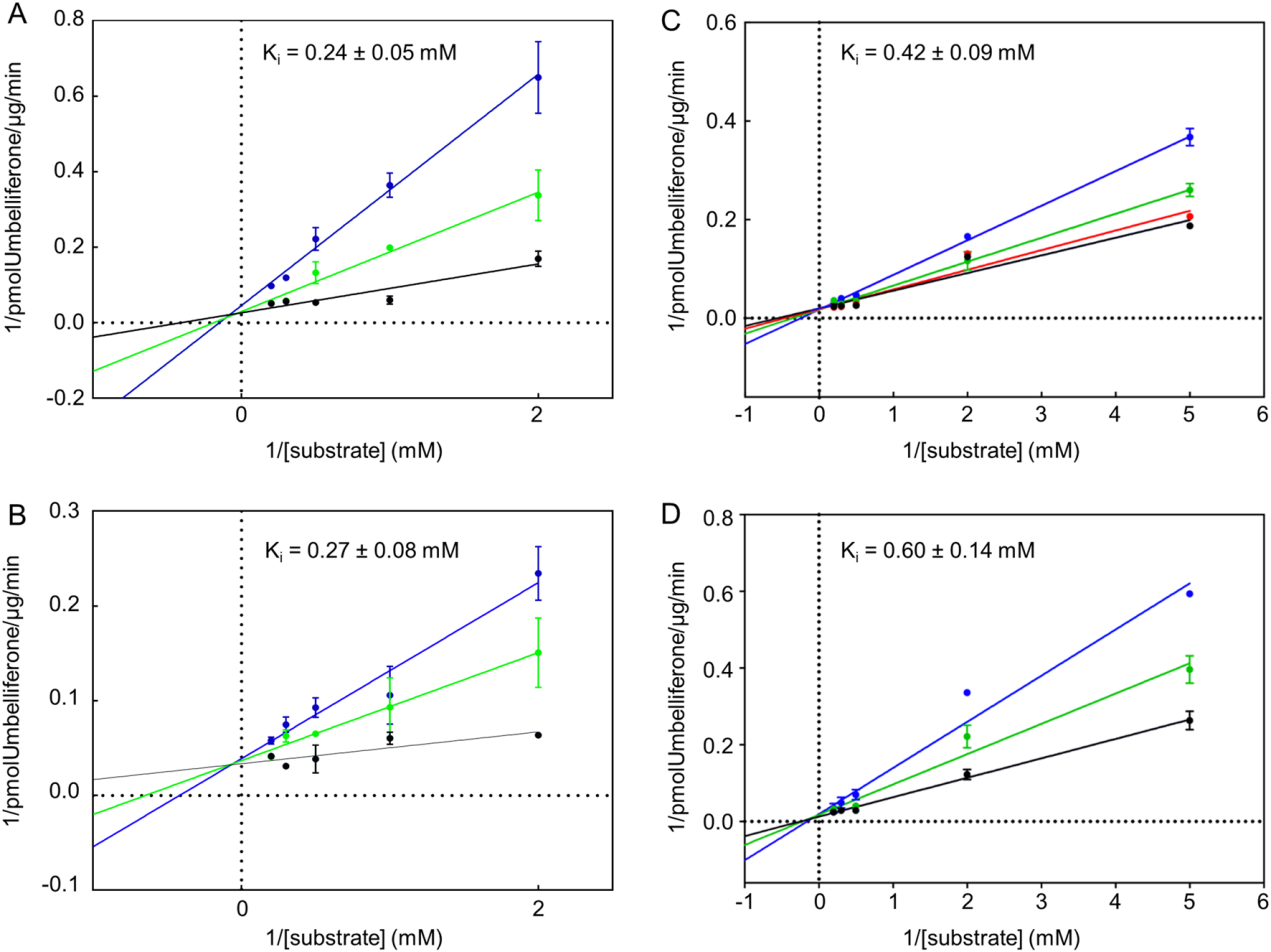
Competitive inhibition of compound 17 and 33 on hSPL and AfuSPL. The graphs show the Lineweaver–Burk plots of the enzymatic activity measured for (**A,C**) hSPL and (**B,D**) AfuSPL as a function of substrate concentrations in presence of different C17 (**A,B**) and C33 (**C,D**) concentrations. The color code is: black, 0 mM; red, 0.1 mM; green, 0.25 mM and blue 1 mM.

We investigated the interaction of compounds 17 and 33 with hSPL and AfuSPL in order to define the inhibition mechanism. By determining the kinetic parameters for the fluorogenic substrate in the presence of each inhibitor at increasing concentrations, we observed a behavior typical of a competitive inhibition. From a global fitting of the data we estimated their K_I_ values, as reported in Fig. 6.

**Figure 7:**
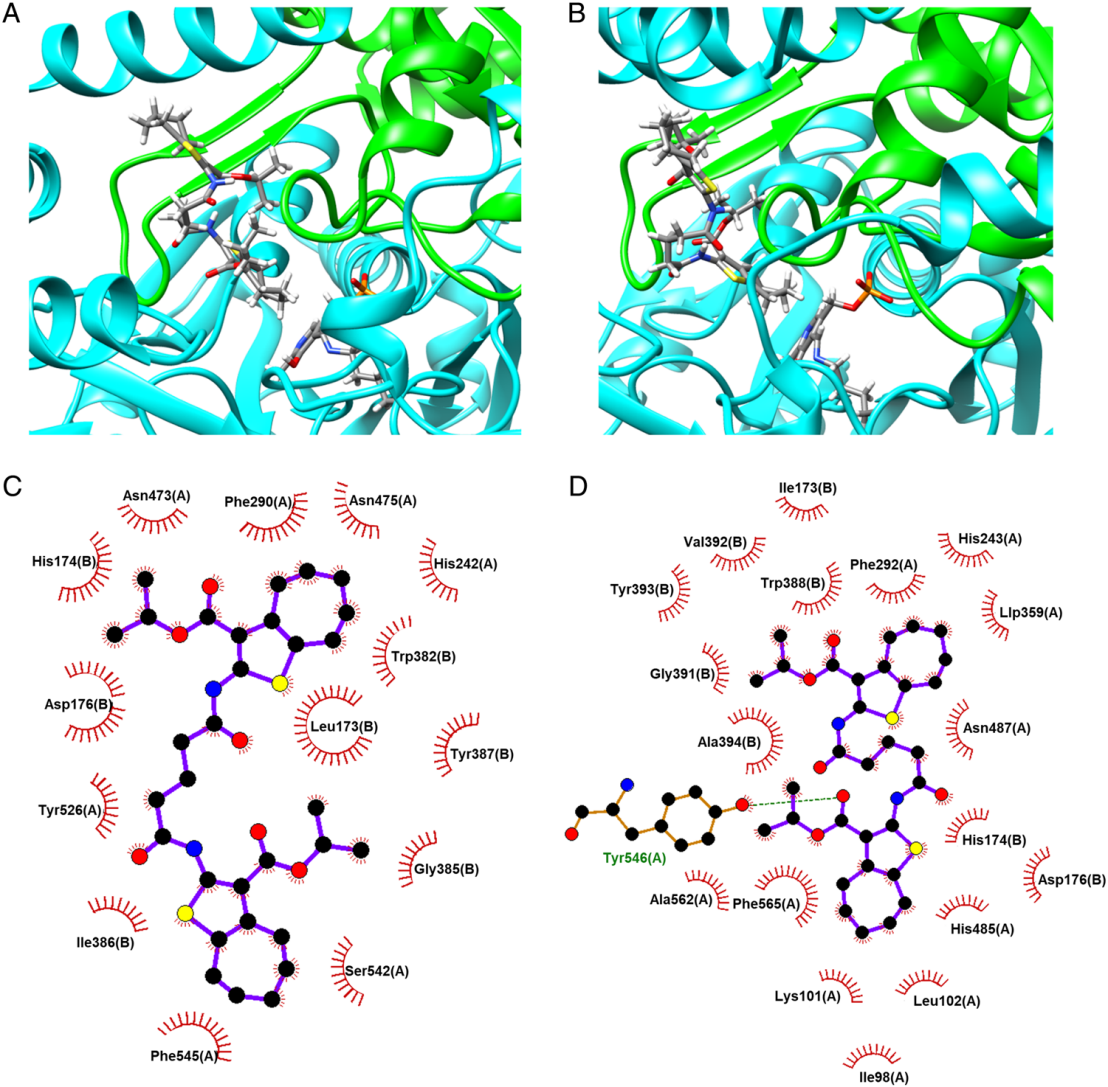
(**A**,**B**) Predicted binding modes of the dual-target ligand C17 in hSPL (**A**) and AfuSPL (**B**) binding sites. Chains A and B of SPL enzymes are displayed as cyan and green cartoons, respectively; C17 ligands and PLP cofactors are shown in sticks (colors by element type). (**C**,**D**) 2D diagrams of ligand–protein interactions are depicted in panels C and D: C17-hSPL (**C**) and C17-AfuSPL (**D**), respectively. Aminoacids hydrophobically interacting with C17 are highlighted; the dashed line indicates the hydrogen bond.

On the basis of its chemical and biological properties, C17 was selected for docking studies to gather information on the molecular interactions between the hit and the two crystallizated SPL proteins, as highlighted in Fig. 7. Indeed, when C17 was docked in the catalytic pocket of both hSPL and AfuSPL, the best predicted poses were in the entrance of the narrow and hydrophobic channel (Fig. 7A, B). The binding is stabilized by several hydrophobic interations (Fig. 7B, C), although AfuSPL also shows a putative hydrogen bond formed by the carbonyl group of the amide function of C17 and the phenolic hydroxyl group of Tyr546(A) (Fig. 7D). The two symmetrical tetrahydrocyclohepta[*b*]thienyl moiety heads of C17 are directed toward the PLP on one side, and the tunnel entrance on the other side. In both enzymes, one of the aromatic thienyl rings of C17 stabilizes the binding by π-stacking with Phe545(A) and Phe565(A) of hSPL and AfuSPL, respectively. Remarkably, C17 fits in the two SPL binding sites without directly interacting with the polar amino acids of the catalytic machinery and the polar PLP cofactor.

## Discussion

The results presented in this study provide the rationale and the experimental basis for the development of SPL inhibitors targeting both human and *Aspergillus* orthologues and working as antifungal and anti-inflammatory drugs in CF. Despite the major breakthrough of Elexacaftor, Tezacaftor and Ivacaftor (ETI) in the pulmonary improvement of CF patients^57^, the relationship between Highly Effective Modulator Therapy (HEMT) and inflammation remains disputed^58^. In addition, while the use of ETI has shown a rapid reduction of infections and antibiotic use^59^, previous studies on single modulator therapy have shown that the initial decrease in bacterial burden is followed by resurgence of bacteria in the lungs after one year, likely because of pre-existing irreversible lung damage^60^. It is unclear whether the same resurgence would occur with ETI and/or upon therapy initiation before lung damage, and the possibility of pathogen persistence in the long period remains a critical issue. The complex relationships between ETI, inflammation and infection, the incomplete genetic spectrum of mutations approved for treatment, the confounding effect of pre-existing lung damage as well as the risk of side effects and drug-drug interactions, make the study of antimicrobial and anti-inflammatory agents a relevant and active field of research in the HEMT era. In this direction, we searched for drug targets that could cooperatively bring together antimicrobial and anti-inflammatory activities, thus bridging resistance and tolerance under the same therapeutic strategy. Indeed, by weakening microbial pathogenicity on the one hand, and limiting immune-mediated host damage on the other hand, this strategy would optimize the overall response and, at the same time, alleviate the high treatment burden of CF patients. Our background research identified S1P, and the related degradative enzyme SPL, as a potential candidate in the context of CF aspergillosis.

SPL, as key enzyme at the exit gate of the S1P degradation pathway, is considered an important drug target to modulate the levels of S1P in a variety of human diseases related to autoimmunity and inflammation including multiple sclerosis and rheumatoid arthritis^20,61^. In addition, the increase in S1P and sphingosine levels resulting from SPL inhibition is also considered a good antimicrobial strategy, in particular for respiratory infections^62^. In agreement with SPL being a well conserved protein from bacteria to humans^19^, we found that hSPL and AfuSPL show very similar biochemical properties, although we could notice differences related to (i) an higher propensity of the fungal enzyme to bind the hydrophobic probe ANS, which may also correlate with the higher aggregation tendency and reduced purification yields, and (ii) some changes in the spectral and kinetic features, probably due to subtle differences in the region surrounding the active site. In this regard, the crystal structures of the two proteins in the ligand-free form confirm that the coenzyme binding regions are superimposable, thus further supporting the possibility to develop a dual-species inhibitor. However, the regions flanking the access to the substrate binding cleft are clearly endowed with a different structural flexibility. In particular, the N-terminus helix, that is structured in AfuSPL and disordered in hSPL, partially covers the access to the binding poket and provides additional hydrophobic residues to stabilise the binding of lipophilic compounds, and this may account for the higher interaction of AfuSPL with ANS. On the other hand, the same helix may reduce the access to the cleft (the calculated cleft volume is 3230 Å^3^ for hSPL compared to 2630 Å^3^ for AfuSPL^63^), and this may explain the lack of inhibition by a hSPL-specific ligand against AfuSPL. The latter finding must be taken into consideration during the drug-discovery process. Indeed, we perfomed a first-round screening campaign aimed at setting the ground for the development of specific ligands targeting both hSPL and AfuSPL.

Our campaign identified a series of lipophilic molecules structurally unrelated to S1P, in agreement with previous reports^64^. Although the screening was performed starting from the available structure of hSPL, we found that most putative ligands were more effective as inhibitors of AfuSPL. A clear explanation to these data is not currently available, but we could speculate that this is due to either the structured N-terminal helix in AfuSPL, which could increase binding affinity by partly closing the cleft and preventing ligand dissociation, or to the increased flexibility of the regions allowing the access to the active site in AfuSPL, which could favour the adaptation of the ligands to the active site cleft. It remains that compounds leading to complete inhibition of the fungal enzyme with selectivity versus the human counterpart merit future investigations as putative anti-fungal agents. Among compounds showing a statistically significant inhibitory activity on both proteins, we chose to focus our attention on C17 and C33 and we were able to verify that they behave as competitive inhibitors, in line with the predicted binding mode. It must be noticed that their K_i_ for both enzymes are in the high micromolar range, a value significantly higher than those of the hSPL inhibitors previously published^20^. Indeed, the reported K_m_ values of human and microbial SPL for the physiological substrate S1P are in the range of 15–20 μM^65,66^. Nevertheless, compounds 17 and 33 are able to compete with the synthetic substrate used in our assay showing a K_m_ in the millimolar range. The competitive effect is corroborated also by docking studies where the predicted pose of the selected inhibitor C17 is located at the entrance of the catalytic channel where the substrate S1P fits in both enzymes. Nonetheless, the low binding affinity for both targets implies that another round of VS followed by a rationale optimization of the best candidate will be necessary before testing in in vitro and in vivo disease models. In this regard, a rational hit-to-lead optimization must be undertaken, based on the structural information available on the ligand-free enzyme, and in particular the putative positioning of the active site cleft with respect to the ER membrane, followed by a medium-throughput screening of ligand–protein interaction through biophysical techniques. In particular, since the natural SPL substrate is amphipatic, and on the catalytic channel are present some polar residues, as depicted in Fig. S7, the synthesis of novel derivatives bearing polar side chains in one of the two symmetrical cyclopentane[*b*]thiophene scaffolds of C17 could give the double advantage of improving target affinity and solubility.

Another aspect to be considered is that S1P is a signalling lipid involved in a variety of physiological and pathological processes by functioning not only inside cells, but also by binding to five S1P receptors^67,68^. This plethora of activities argues for caution when attempting to modulate its levels. For instance, inhibiting SPL to increase S1P may be beneficial to inhibit lymphocyte trafficking in autoimmune diseases or promoting cell survival in conditions such as lung injury or cardiac damage by ischemia/reperfusion, but may expose to pulmonary fibrosis, increased risks of cancerogenesis or impaired immune functions. Conversely, SPL overexpression to reduce S1P may be beneficial in cancer, but at the same time may sensitize non-malignant cells to cytotoxic therapy^69^. It is also becoming increasingly clear by the clinical picture of SPL insufficiency syndrome (SPLIS) patients that SPL in involved in several physiologic functions in kidney, immune system (lymphocyte and neutrophil trafficking), skin, neurological system, and adrenal gland^70^, thus arguing for caution in targeting SPL. As a way to overcome potential side effects of SPL modulation, local drug delivery would specifically increase S1P levels only where it is needed. This is particularly relevant in CF. Indeed, it has been recently reported that serum S1P levels are not significantly different between CF patients and healthy controls, and are further increased by ETI therapy^71^. In addition, lung delivery can help to overcome possible pharmacokinetic issues related to scarce solubility or off target interactions. In fact, the pulmonary route is known to enable high local drug levels, while reducing dose as well as systemic side effects. This strategy may achieve efficacy even in case of non-optimal target affinity. It would be therefore important to restrict SPL inhibition to the lung where S1P levels are decreased. This approach has already been applied by our group to deliver anti-inflammatory agents directly into the lungs in a CF setting to optimize the efficacy/safety profile^72–74^.

In conclusion, in this study we provide evidence that the development of dual species SPL inhibitors with potential combined antifungal and anti-inflammatory activities in CF is a feasible therapeutic strategy. Structure-based hit optimization and pharmaceutical formulations for localized lung delivery will pave the way for the translational development of potential drugs in the perspective of a combination therapy with HEMT to integrate CFTR correction and potentiation with immune and microbial modulating activities, at least in the context of aspergillosis.

### Supplementary Information

Supplementary Information.

## Data Availability

The datasets generated during and/or analysed during the current study are available from the corresponding author on reasonable request.

## Abbreviations

CF: Cystic fibrosis
S1P: Sphingosine-1-phosphate
Cer: Ceramide
SPL: Sphingosine phosphate lyase
PLP: Pyridoxal 5′-phosphate
AfuSPL: SPL from *Aspergillus fumigatus*
hSPL: Human SPL
CD: Circular dichroism
