# Supplementary material for: Dual species sphingosine-1-phosphate lyase inhibitors to combine antifungal and anti-inflammatory activities in cystic fibrosis: a feasibility study
Source: Sci Rep. 2023 Dec 20;13:22692. doi: 10.1038/s41598-023-50121-4 (PMC10733307; doi:10.1038/s41598-023-50121-4)
Supplement: Supplementary file 1 — Supplementary Information. [file 41598_2023_50121_MOESM1_ESM.pdf]

DUAL SPECIES SPHINGOSINE-1-PHOSPHATE LYASE INHIBITORS TO COMBINE ANTIFUNGAL AND ANTI-INFLAMMATORY ACTIVITIES IN CYSTIC FIBROSIS: A FEASIBILITY STUDY

Barbara Cellini[1,#*], Gioena Pampalone[1*], Emidio Camaioni[2,**], Marilena Pariano[1,**], Flavia Catalano[3], Teresa Zelante[1], Mirco Dindo[1], Lara Macchioni[1], Alessandra Di Veroli[4], Roberta Galarini[5], Fabiola Paoletti[5], Magdalena Davidescu[1], Claudia Stincardini[1], Gianluca Vascelli[1], Marina Maria Bellet[1], Julie Saba[6], Stefano Giovagnoli[2], Giorgio Giardina[3], Luigina Romani[1], Claudio Costantini[1,#]

[1]Department of Medicine and Surgery, University of Perugia, Perugia, Italy

[2]Department of Pharmaceutical Sciences, University of Perugia, Perugia, Italy

[3]Department of Biochemical Sciences "A. Rossi Fanelli", Sapienza University of Rome, Rome, Italy

[4]Department of Chemistry, Biology and Biotechnology, University of Perugia, Perugia, Italy.

[5]Centro Sviluppo e Validazione Metodi, Istituto Zooprofilattico Sperimentale dell'Umbria e delle Marche "Togo Rosati", Perugia, Italy

[6]Department of Pediatrics, University of California San Francisco, San Francisco, CA

SUPPLEMENTARY INFORMATION

Table S1 - AutoDockVina docking parameters

Parameters		hSPL – 8AYF	AfuSPL – 8CMX
Searching box coordinates	Center_x	12.0	12.0
	Center_y	-49.5	10.0
	Center_z	-19.0	-9.0
Searching box Dimensions (Å)	Size_x	25	25
	Size_y	25	25
	Size_z	25	25
Number of poses		10	10
Energy range (Kcal)		3	3
Exhaustiveness		64	64
Explicit seed		1234	1234

Table S2 – Crystallization, data collection, model building and refinement statistics

	**hSPL - 8AYF**	**AfuSPL - 8CMX**
**Crystallization**		
Vapour diffusion method	Sitting drop	Sitting drop
Protein concentration (µM)	108	93
Protein buffer	Tris-HCl 20 mM pH 8.0; NaCl 150 mM; DTT 1mM; Glycerol 5%	Tris-HCl 20 mM pH 8.0; NaCl 150 mM; DTT 1mM; Glycerol 5%; 1% DMSO; 1 mM compound 33
Reservoir composition	0.02M Sodium formate; 0.02M Ammonium acetate; 0.02M Sodium citrate tribasic dihydrate; 0.02M Potassium sodium tartrate tetrahydrate; 0.02M Sodium oxamate; Imidazole 0.05M; MES monohydrate (acid) 0.05M; 12.5% v/v MPD; 12.5% PEG 1000; 12.5% w/v PEG 3350	0,1 M Hepes sodium pH 7,5; 1.4 M Sodium citrate tribasic dihydrated
Drop setup (µL)	0.4 + 0.4	0.3 +0.3
Temperature (°C)	21	21
Crystal growth time (d)	14	60
Cryoprotection	none	Reservoir + glycerol 20%
**Data collection**		
Beamline	Elettra XRD2	Elettra XRD2
Wavelength	1.000	1.000
Resolution range	57.70 - 1.84 (1.906 - 1.84)	101.50 - 3.46 (3.74 - 3.46)
Space group	P 1 21 1	P 65 2 2
Unit cell (Å,degrees)	59.16 127.41 66.95<br>90 104.85 90	130.07 130.07 234.06<br>90 90 120
Unique reflections	47601 (2380)	12600 (631)
Multiplicity	6.2 (6.1)	36.7 (39.0)
Completeness ellipsoidal (%)	92.6 (67.4)	95.1 (71.9)
Mean I/sigma(I)	7.8 (1.7)	15.1 (1.7)
Wilson B-factor ($Å^2$)	18.8	113.7
CC1/2	0.99 (0.42)	0.99 (0.57)
**Model building and refinement**		
R-work	0.191 (0.281)	0.284 (0.405)
R-free	0.215 (0.421)	0.296 (0.422)
Number of non-hydrogen atoms	7240	7371
Protein atoms	6892	7343
ligands (PLP)	28	28

solvent	320	0
Protein residues	897	974
RMS (bonds, Å)	0.016	0.008
RMS (angles, degrees)	1.55	0.89
Ramachandran favored (%)	96.6	93.8
Ramachandran allowed (%)	3.4	5.9
Ramachandran outliers (%)	0	0.3
Rotamer outliers (%)	3.5	0.3
Clashscore	9.91	14.32
Average B-factor (all model) ($Å^2$)	24.9	123
Protein residues (all atoms)	24.8	123.3
ligands (PLP)	21.9	77.6
solvent	28.7	-

Values in parenthesis refer to higher resolution shell.

Table S3. Compounds from the SPECS vendor.

#	SPECS code	Smiles
C1	AF-399/41767596	COc1ccc(cc1)NC(N2CCN(CC2)c3ccccn3)=S
C2	AG-205/33116027	O=C(c1ccc2C(=O)N(C(=O)c2(c1))N3C(=O)c4ccccc4(C3(=O)))c5ccc6C(=O)N(C(=O)c6(c5))N7C(=O)c8ccccc8(C7(=O))
C3	AG-690/33095035	CCCCCCCCCCNS(=O)(=O)c3cc2C(=O)c1cc(cc(c1c2c(c3)[N+](=O)[O-])[N+](=O)[O-])S(=O)(=O)NCCCCCCCCCC
C4	AG-205/33128033	CCOc2cc(ccc2(OCc1ccccc1))C=NNC(=O)C(CSCc3ccccc3)NC(=O)c4cc(cc(c4)[N+](=O)[O-])[N+](=O)[O-]
C5	AG-690/37079104	O=C4Nc1ccccc1C4(=NN2CCN(CC2)c3ccccn3)
C6	AG-690/37012141	Cc1ccc2cc(C)c(nc2(c1))N3CCN(CC3)c4ccccn4
C7	AG-690/36169064	O=C3c4ccc5C(=O)N(c1cccc(c1)Cl)C(=O)c6c(cc(C(=O)N3(c2cccc(c2)Cl))c4c56)Nc7cccc(c7)Cl
C8	AG-670/08601012	Cc1ccc(cc1)C67(CC5CC(CC(CNC(=O)c2ccc(cc2)N=Cc3ccc(cc3)c4ccc(cc4)OC(C)=O)(C5)C6)C7)
C9	AG-690/34441024	O=C1OC(=Nc2ccccc12)c8ccc9C(=O)N(c3ccc(cc3)N4C(=O)c5ccc(cc5(C4(=O)))C6=Nc7ccccc7(C(=O)O6))C(=O)c9(c8)
C10	AG-205/07900024	Cc1ccc(cc1(C))NC(=O)c2ccc(cc2)N3C(=O)c4ccc(cc4(C3(=O)))C(=O)Nc5ccc(C)c(C)c5
C11	AG-690/11352198	O=C8c1cccc(c1C(=O)N8(c2ccc(cc2)c4nc3ccc(cc3c(n4)c5ccccc5)N7C(=O)c6cccc(c6C7(=O))Cl))Cl
C12	AC-776/41252551	c1ccc2c(c1)cccc2c8ccc(c4ccc(c3ccccc34)c7ccc(c6cccc5ccccc56)s7)s8
C13	AG-690/36538051	O=[N+]([O-])c1ccc(cc1[N+](=O)[O-])c3nc2ccccc2c(n3)c4ccc(cc4)c6nc(nc5ccccc56)c7ccc(c(c7)[N+](=O)[O-])[N+](=O)[O-]
C14	AK-918/12272195	O=C4C8C(C(=O)N4(c1ccc(cc1)Oc2ccc3ccccc3(c2)))C7(c5ccccc5C8(c6ccccc67)Br)Br
C15	AK-918/12272196	O=C7C8C3c1ccccc1C(c2ccccc23)C8(C(=O)N7(c4ccc(cc4)Oc5ccc6ccccc6(c5)))
C16	AG-690/12892641	CC3=CC(C)(C)N(C(=O)c1ccc(cc1)c2ccccc2)c4ccc(cc34)OC(=O)c5ccc(cc5)c6ccccc6
C17	AK-968/13031184	CCCOC(=O)c4c1CCCCCc1sc4(NC(=O)CCCC(=O)Nc3c(C(=O)OCCC)c2CCCCCc2s3)
C18	AN-465/14458072	O=[N+]([O-])c1cc(ccc1N2CCN(CC2)c3ccc(nn3)Cl)C(F)(F)F
C19	AM-900/15248098	CCOC(=O)C5=C(C)N=C3N(C(=O)C(=Cc1ccc(cc1)OS(=O)(=O)c2ccc(C)cc2)S3)C5(c4ccc(OC)c(c4)OC)
C20	AM-900/15548270	O=C(Cn5cc(C=NNc1nc(nc(n1)N2CCCC2)N3CCCC3)c4ccccc45)n8c6ccccc6sc7ccc(cc78)Cl
C21	AF-399/40654538	Cc1ccc(cc1)c2csc3ncnc(c23)N4CCN(CC4)c5ccccn5
C22	AN-970/40920574	c1ccc(cc1)C6=CC(=NC6(=Nc3[nH]c(cc3(c2ccccc2))c5cccc4ccccc45))c8cccc7ccccc78
C23	AK-968/41025915	CCOC(=O)C5=C(C)N=C3N(C(=O)C(=Cc2ccc(COc1ccc(cc1[N+](=O)[O-])Cl)o2)S3)C5(c4ccccc4)
C24	AK-918/41698622	O=S(=O)(Nc2cccc1ccccc12)c4ccc5oc3ccc(cc3c5(c4))S(=O)(=O)Nc7cccc6ccccc67

C25	AF-399/41346082	CCOC(=O)c5c(C)c(C)sc5(NC(=O)CSc4nnc(CCCCCN1C(=O)c3cccc2cccc(C1(=O))c23)o4)
C26	AN-329/40920358	CNC(=O)c2ccccc2(NC(=O)c1ccc(cc1)C(C)(C)C)
C27	AN-329/40922901	CCOc1ccc(cc1)NS(=O)(=O)c2ccc(cc2)NC(=O)CCCCC(=O)Nc3ccc(cc3)S(=O)(=O)Nc4ccc(cc4)OCC
C28	AN-329/40869134	Cc4cc(C)c(NS(=O)(=O)c1ccc(cc1)NC(=O)CCCCC(=O)Nc2ccc(cc2)S(=O)(=O)Nc3c(C)cc(C)cc3(C))c(C)c4
C29	AN-329/13484046	O=C(CCCCCCCC(=O)Nc1ccc(cc1)S(=O)(=O)N3CCc2ccccc23)Nc4ccc(cc4)S(=O)(=O)N6CCc5ccccc56
C30	AP-406/41885709	c1ccc(cc1)c3cc2ccc6cc2c(n3)c5nc(cc4ccc(cc45)OCc7cccc(CO6)c7)c8ccccc8
C31	AH-487/40936941	COc1ccc(cc1)N(CC(=O)Nc2ccc(cc2)CSc3ccccc3)S(=O)(=O)c4ccc(C)c(c4)[N+](=O)[O-]
C32	AK-968/41024878	CCOC(=O)C5=C(C)N=C3N(C(=O)C(=Cc2ccc(COc1ccc(F)cc1Cl)o2)S3)C5(c4ccccc4)
C33	AK-968/41024883	CCOC(=O)C5=C(C)N=C3N(C(=O)C(=Cc2ccc(COc1ccc(F)c(c1)Cl)o2)S3)C5(c4ccccc4)
C34	AK-968/41024966	CCOC(=O)C5=C(C)N=C3N(C(=O)C(=Cc2ccc(COc1ccc(F)cc1(F))o2)S3)C5(c4ccccc4)
C35	AK-968/41017666	COc1ccc(cc1)C5C(=C(C)N=C4N5(C(=O)C(=Cc3ccc(COc2cccc(c2)C(F)(F)F)o3)S4))C(=O)OC
C36	AK-968/41171897	COc1ccc(cc1)C5C(=C(C)N=C4N5(C(=O)C(=Cc3ccc(COc2cccc(c2Cl)Cl)o3)S4))C(=O)OC
C37	AK-968/41172083	COC(=O)C5=C(C)N=C3N(C(=O)C(=Cc2ccc(COc1cccc(c1Cl)Cl)o2)S3)C5(c4ccccc4)
C38	AC-776/41252526	c1ccc2c(c1)cccc2c6ccc(c5cccc3c5(cccc3c4cccs4))s6
C39	AK-968/41172081	CCOC(=O)C5=C(C)N=C3N(C(=O)C(=Cc2ccc(COc1cccc(c1Cl)Cl)o2)S3)C5(c4ccc(cc4)N(C)C)
C40	AK-968/41923279	COC(=O)C5=C(C)N=C3N(C(=O)C(=Cc2ccc(COc1ccc(F)cc1(F))o2)S3)C5(c4ccccc4)
C41	AK-968/41923302	COC(=O)C5=C(C)N=C3N(C(=O)C(=Cc2ccc(COc1cc(F)ccc1[N+](=O)[O-])o2)S3)C5(c4ccccc4)
C42	AK-968/41923369	COC(=O)C5=C(C)N=C3N(C(=O)C(=Cc2ccc(COc1ccc(cc1[N+](=O)[O-])Cl)o2)S3)C5(c4ccccc4)
C43	AK-968/41024910	CCOC(=O)C5=C(C)N=C3N(C(=O)C(=Cc2ccc(COc1cccc(c1)C(F)(F)F)o2)S3)C5(c4ccccc4)
C44	AK-968/41025914	CCOC(=O)C5=C(C)N=C3N(C(=O)C(=Cc2ccc(COc1cc(ccc1(F))[N+](=O)[O-])o2)S3)C5(c4ccccc4)
C45	AK-968/41923244	COC(=O)C5=C(C)N=C3N(C(=O)C(=Cc2ccc(COc1ccc(F)c(c1)Cl)o2)S3)C5(c4ccccc4)
C46	AC-776/15493185	COc1ccc(cc1)c5ccc(c2cc(OC)c(cc2(OC))c4ccc(c3ccc(cc3)OC)s4)s5
C47	AC-776/15493186	c1ccc(cc1)c5ccc(c2cccc(c2)c4ccc(c3ccccc3)s4)s5
C48	AN-758/13190033	CCOC(=O)C5=C(C)N=C3N(C(=O)C(=Cc1ccc(cc1)OCc2ccccc2[N+](=O)[O-])S3)C5(c4ccccc4)
C49	AN-758/13190040	CCOC(=O)C5=C(C)N=C3N(C(=O)C(=Cc1ccc(OC)c(c1)OCc2ccc(cc2)Cl)S3)C5(c4ccccc4)

C50	AK-968/41024934	CCOC(=O)C5=C(C)N=C3N(C(=O)C(=Cc2ccc(COc1cccc(c1Cl)Cl)o2)S3)C5(c4ccccc4)

Table S4. Statistical significance related to Figure 5 and S4. One-way ANOVA.

hSPL				
**Dunnett's multiple comparisons test**	**Mean Diff**	**95% CI of diff**	**Significant?**	**Summary**
Negative control vs. C1	2,334	-15,89 to 20,56	No	ns
Negative control vs. C2	1,815	-16,41 to 20,04	No	ns
Negative control vs. C3	9,853	-8,368 to 28,07	No	ns
Negative control vs. C4	6,224	-10,07 to 22,52	No	ns
Negative control vs. C5	18,51	1,420 to 35,61	Yes	*
Negative control vs. C6	4,418	-13,80 to 22,64	No	ns
Negative control vs. C7	8,196	-10,03 to 26,42	No	ns
Negative control vs. C8	4,471	-13,75 to 22,69	No	ns
Negative control vs. C9	12,01	-7,954 to 31,97	No	ns
Negative control vs. C10	4,233	-13,99 to 22,45	No	ns
Negative control vs. C11	18,95	0,7261 to 37,17	Yes	*
Negative control vs. C13	14,35	-2,745 to 31,44	No	ns
Negative control vs. C14	12,66	-7,304 to 32,62	No	ns
Negative control vs. C15	5,885	-12,34 to 24,11	No	ns
Negative control vs. C16	13,59	-6,374 to 33,55	No	ns
Negative control vs. C17	64,58	46,36 to 82,81	Yes	****
Negative control vs. C18	6,392	-11,83 to 24,61	No	ns
Negative control vs. C19	3,875	-14,35 to 22,10	No	ns
Negative control vs. C20	24,43	6,210 to 42,65	Yes	**
Negative control vs. C21	23,84	5,622 to 42,06	Yes	**
Negative control vs. C22	1,436	-16,79 to 19,66	No	ns
Negative control vs. C23	0,0	-18,22 to 18,22	No	ns
Negative control vs. C24	9,278	-8,943 to 27,50	No	ns
Negative control vs. C25	1,313	-16,91 to 19,53	No	ns
Negative control vs. C26	5,646	-14,31 to 25,61	No	ns
Negative control vs. C27	0,0	-18,22 to 18,22	No	ns
Negative control vs. C28	21,50	4,404 to 38,59	Yes	**
Negative control vs. C29	4,004	-14,22 to 22,23	No	ns
Negative control vs. C31	8,840	-9,381 to 27,06	No	ns
Negative control vs. C32	7,336	-10,89 to 25,56	No	ns
Negative control vs. C33	49,98	26,93 to 73,03	Yes	****
Negative control vs. C34	12,61	-5,610 to 30,83	No	ns
Negative control vs. C35	17,28	-0,9364 to 35,51	No	ns
Negative control vs. C36	18,23	0,01327 to 36,46	Yes	*
Negative control vs. C37	2,320	-13,98 to 18,62	No	ns
Negative control vs. C39	6,665	-11,56 to 24,89	No	ns
Negative control vs. C40	3,969	-14,25 to 22,19	No	ns
Negative control vs. C41	0,0	-18,22 to 18,22	No	ns
Negative control vs. C42	12,48	-3,822 to 28,77	No	ns
Negative control vs. C43	15,46	-2,759 to 33,68	No	ns
Negative control vs. C44	1,976	-16,24 to 20,20	No	ns
Negative control vs. C45	23,98	5,759 to 42,20	Yes	**
Negative control vs. C47	15,55	-4,413 to 35,51	No	ns
Negative control vs. C48	6,076	-10,22 to 22,37	No	ns
Negative control vs. C49	6,414	-11,81 to 24,64	No	ns
Negative control vs. C50	1,829	-14,47 to 18,13	No	ns

AfuSPL				
Dunnett's multiple comparisons test	Mean Diff	95% CI of diff,	Significant?	Summary
Negative control vs. C1	78,54	65,56 to 91,52	Yes	****
Negative control vs. C2	69,29	57,44 to 81,14	Yes	****
Negative control vs. C3	84,36	72,51 to 96,21	Yes	****
Negative control vs. C4	76,49	64,64 to 88,33	Yes	****
Negative control vs. C5	83,86	72,01 to 95,71	Yes	****
Negative control vs. C6	86,16	73,18 to 99,14	Yes	****
Negative control vs. C7	78,16	65,18 to 91,14	Yes	****
Negative control vs. C8	82,55	69,57 to 95,53	Yes	****
Negative control vs. C9	98,70	87,58 to 109,8	Yes	****
Negative control vs. C10	81,61	69,76 to 93,46	Yes	****
Negative control vs. C11	77,04	64,06 to 90,02	Yes	****
Negative control vs. C13	82,43	69,45 to 95,41	Yes	****
Negative control vs. C14	81,53	69,68 to 93,38	Yes	****
Negative control vs. C15	82,31	69,33 to 95,29	Yes	****
Negative control vs. C16	84,57	73,97 to 95,16	Yes	****
Negative control vs. C17	85,30	72,32 to 98,28	Yes	****
Negative control vs. C18	69,58	56,60 to 82,56	Yes	****
Negative control vs. C19	73,72	61,87 to 85,57	Yes	****
Negative control vs. C20	70,42	57,44 to 83,40	Yes	****
Negative control vs. C21	80,61	69,50 to 91,73	Yes	****
Negative control vs. C22	70,55	57,57 to 83,53	Yes	****
Negative control vs. C23	76,31	65,19 to 87,42	Yes	****
Negative control vs. C24	79,04	66,06 to 92,02	Yes	****
Negative control vs. C25	76,65	63,67 to 89,63	Yes	****
Negative control vs. C26	78,04	66,19 to 89,88	Yes	****
Negative control vs. C27	71,62	58,64 to 84,60	Yes	****
Negative control vs. C28	84,51	72,66 to 96,36	Yes	****
Negative control vs. C29	75,39	63,55 to 87,24	Yes	****
Negative control vs. C31	74,80	62,95 to 86,65	Yes	****
Negative control vs. C32	77,57	64,59 to 90,55	Yes	****
Negative control vs. C33	49,05	34,07 to 64,04	Yes	****
Negative control vs. C34	75,92	64,07 to 87,77	Yes	****
Negative control vs. C35	74,25	62,40 to 86,10	Yes	****
Negative control vs. C36	74,82	62,97 to 86,67	Yes	****
Negative control vs. C37	78,91	68,32 to 89,51	Yes	****
Negative control vs. C39	81,90	70,05 to 93,75	Yes	****
Negative control vs. C40	77,65	65,80 to 89,50	Yes	****
Negative control vs. C41	82,72	70,87 to 94,57	Yes	****
Negative control vs. C42	86,76	74,91 to 98,61	Yes	****
Negative control vs. C43	75,52	63,67 to 87,37	Yes	****
Negative control vs. C44	74,98	62,00 to 87,96	Yes	****
Negative control vs. C45	83,67	70,69 to 96,65	Yes	****
Negative control vs. C47	82,58	69,60 to 95,56	Yes	****
Negative control vs. C48	50,54	38,69 to 62,38	Yes	****
Negative control vs. C49	78,13	66,28 to 89,98	Yes	****
Negative control vs. C50	83,38	71,53 to 95,23	Yes	****

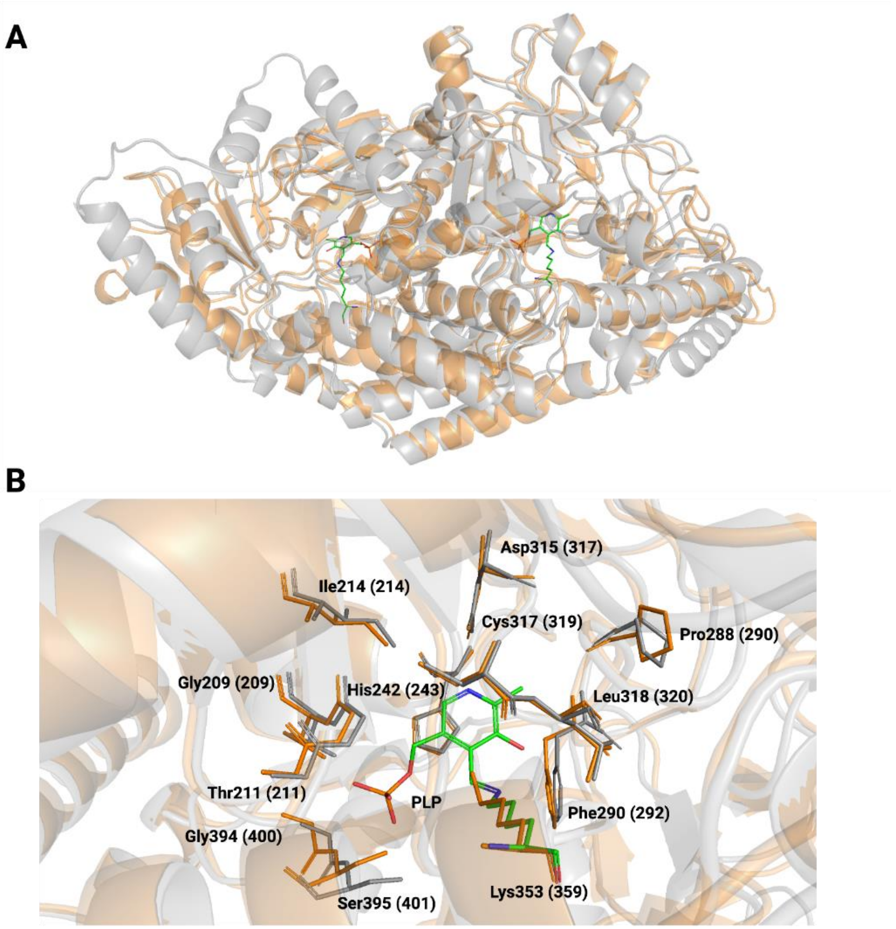

Figure S1. Structure of AfuSPL obtained by homology modelling superimposed with the 3D structure of hSPL. (A) Overall tridimensional structure of hSPL (in grey, PDB code 4Q6R) superimposed with AfuSPL modelled structure (in orange). (C) Representation of the active site residues of hSPL (in grey) and AfuSPL (in orange) within 5 Å from PLP (represented as green sticks).

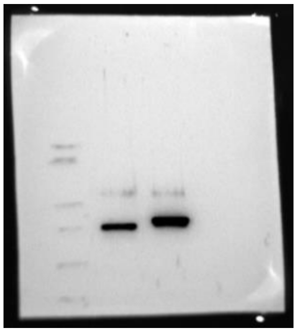

Figure S2. Original membrane of Figure 3A

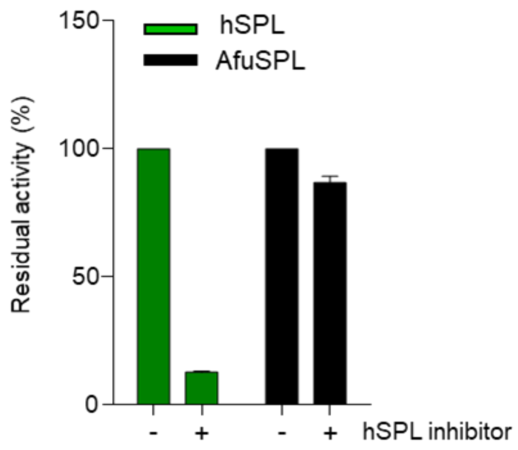

Figure S3. Residual lyase activity of recombinant purified hSPL and AfuSPL in the presence of a known hSPL inhibitor (compound 31 from [20]).

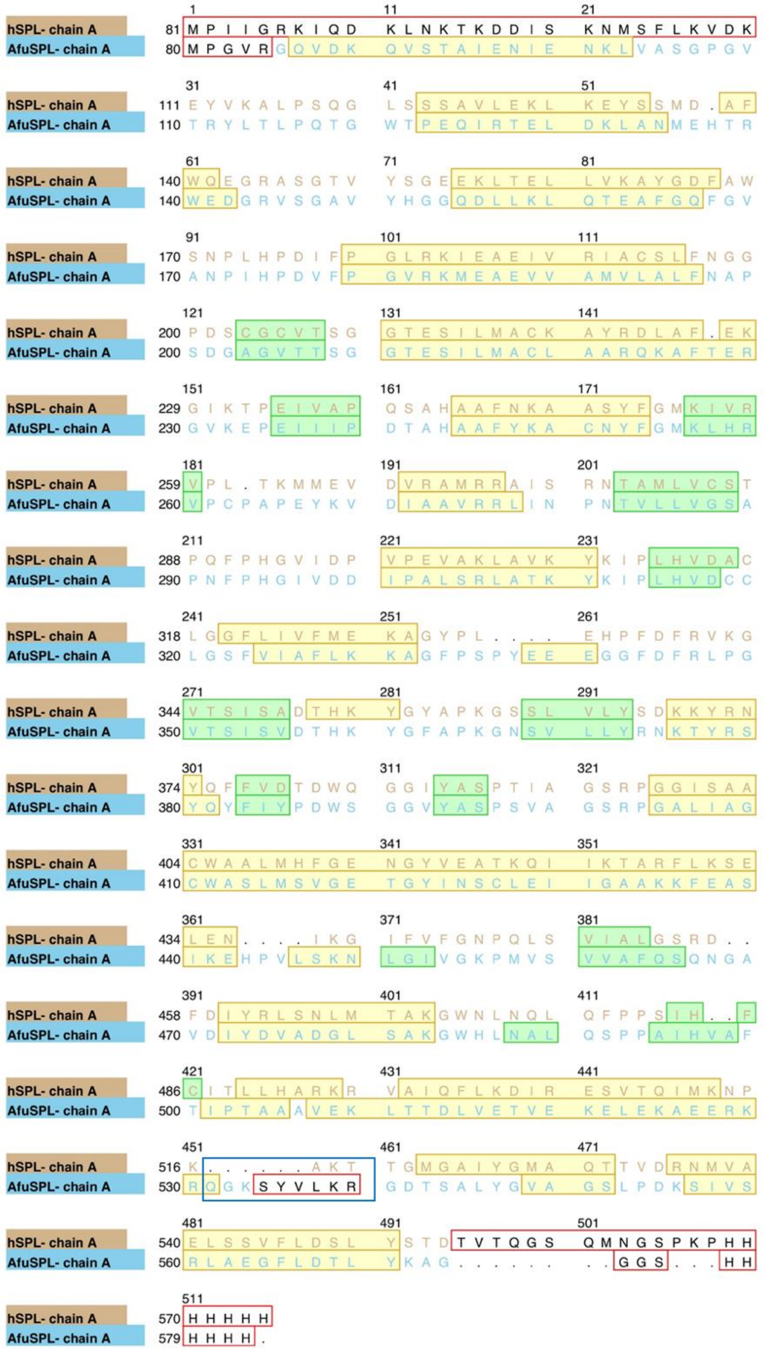

Figure S4. Sequence alignment (including expression tags) for chain A of the crystallised hSPL (gold) and AfuSPL (blue). Secondary structure elements are boxed in yellow (helices) and green (β-strands). Red boxes indicate residues that are not visible in the electron density as they are too flexible and do not assume a defined conformation. Notice that the N-terminus of AfuSPL is almost entirely structured, whereas the same region of hSPL is not. Also notice that the region corresponding to the insertion in AfuSPL (boxed in blue) is also very flexible in hSPL and a stretch of six residues is not visible in the density.

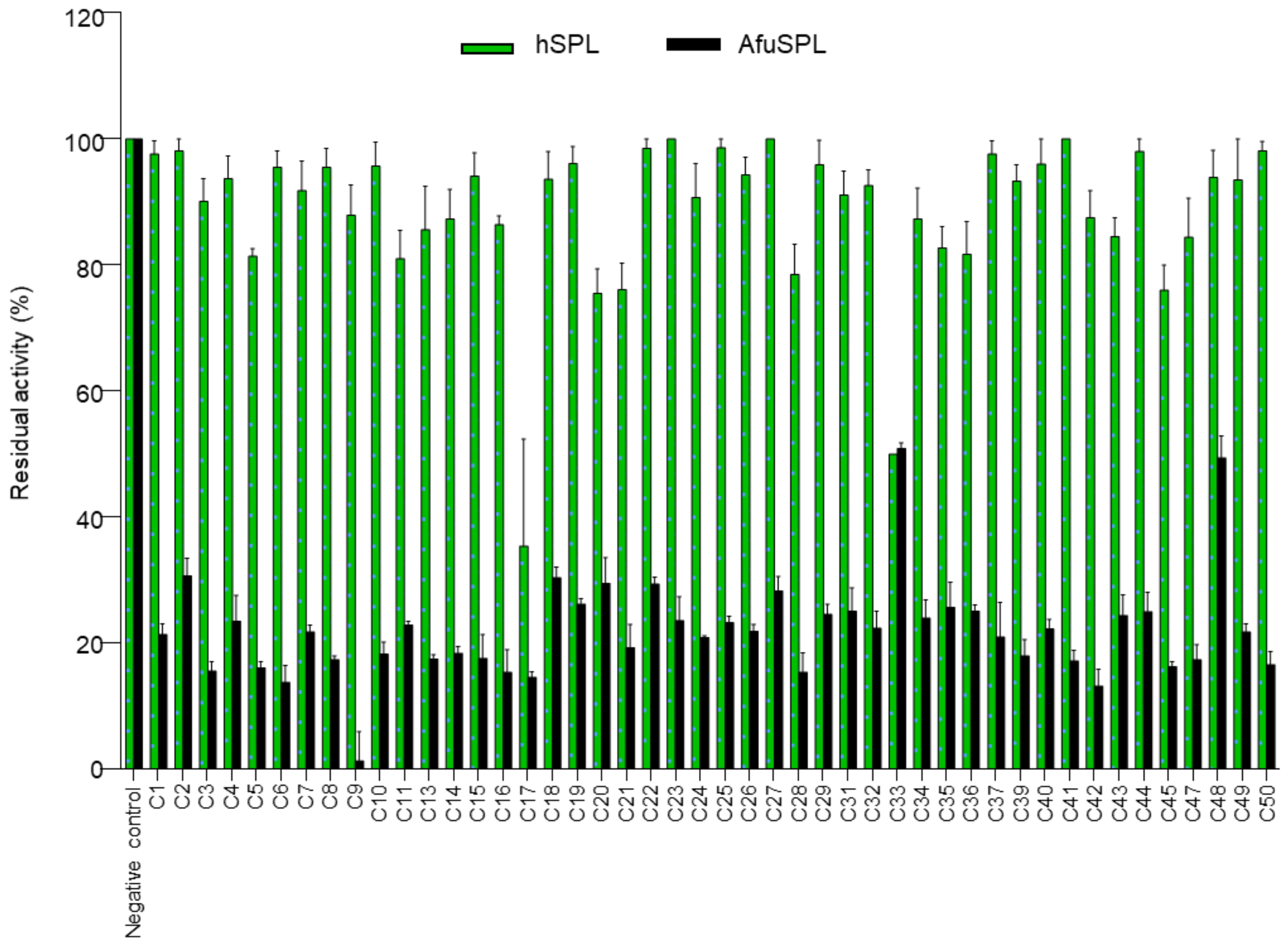

Figure S5. Residual lyase activity of recombinant purified hSPL and AfuSPL in the presence of each candidate selected by the virtual screening campaign at 1 mM concentration. Significance is reported in Table S4.

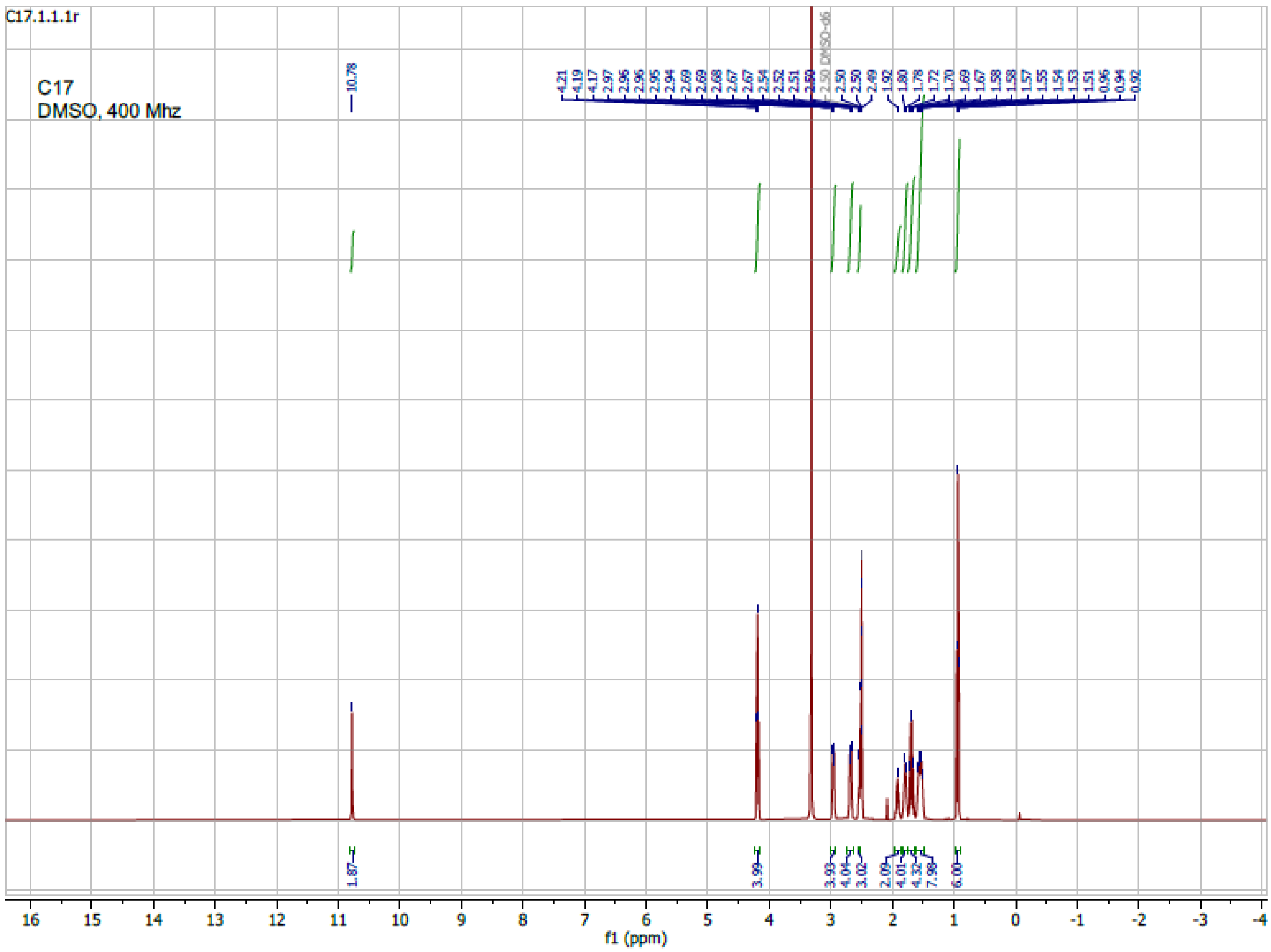
C17.1.1.1r
C17
DMSO, 400 Mhz
f1 (ppm)

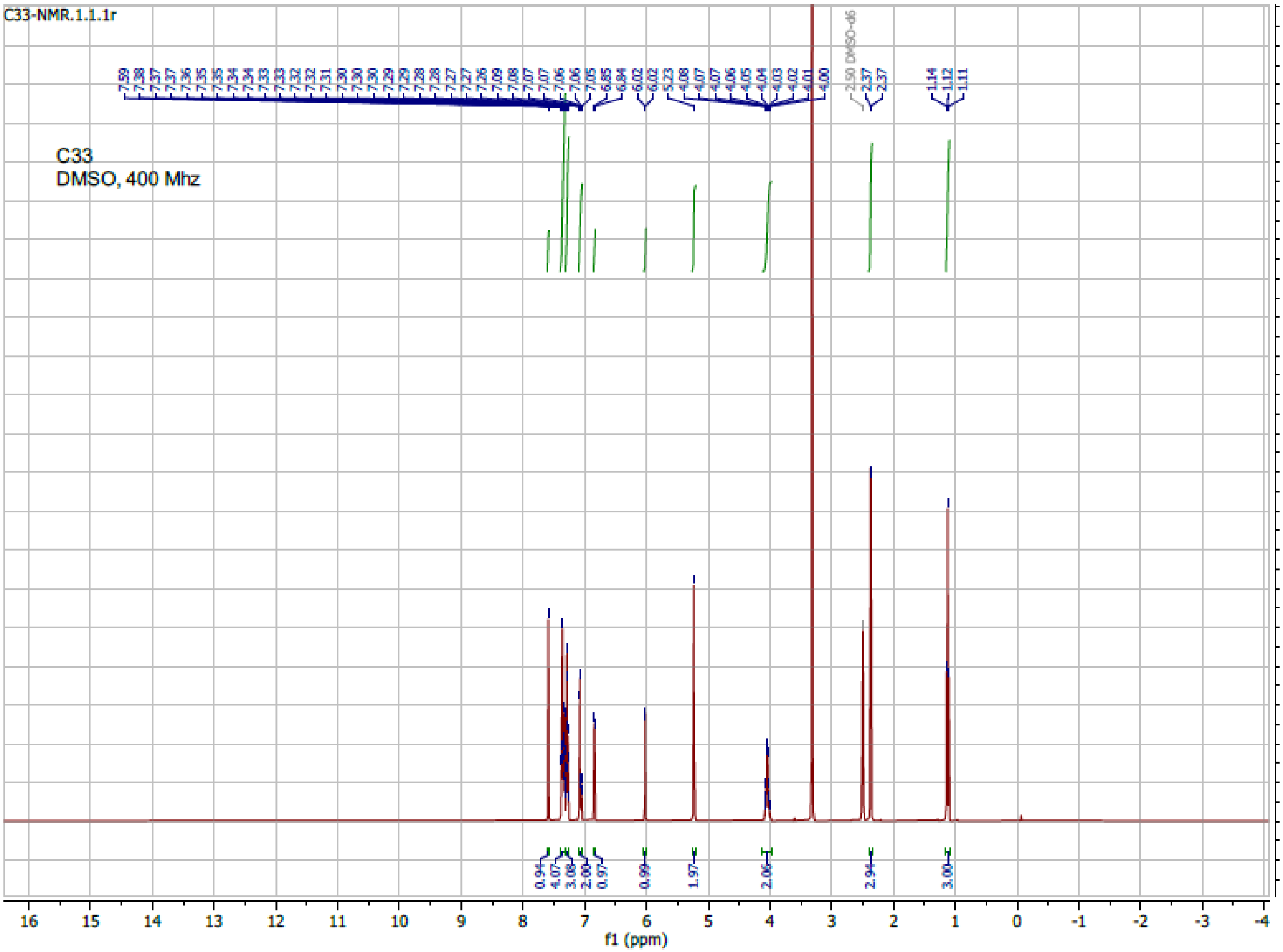

Figure S6. ^{1}H-NMR spectra of C33 and C17. Compounds were dissolved in DMSO-*d*6 and the spectra were recorded at 400 MHz by using a Bruker AC-400 spectrometer. Chemical shifts are reported in ppm (δ).

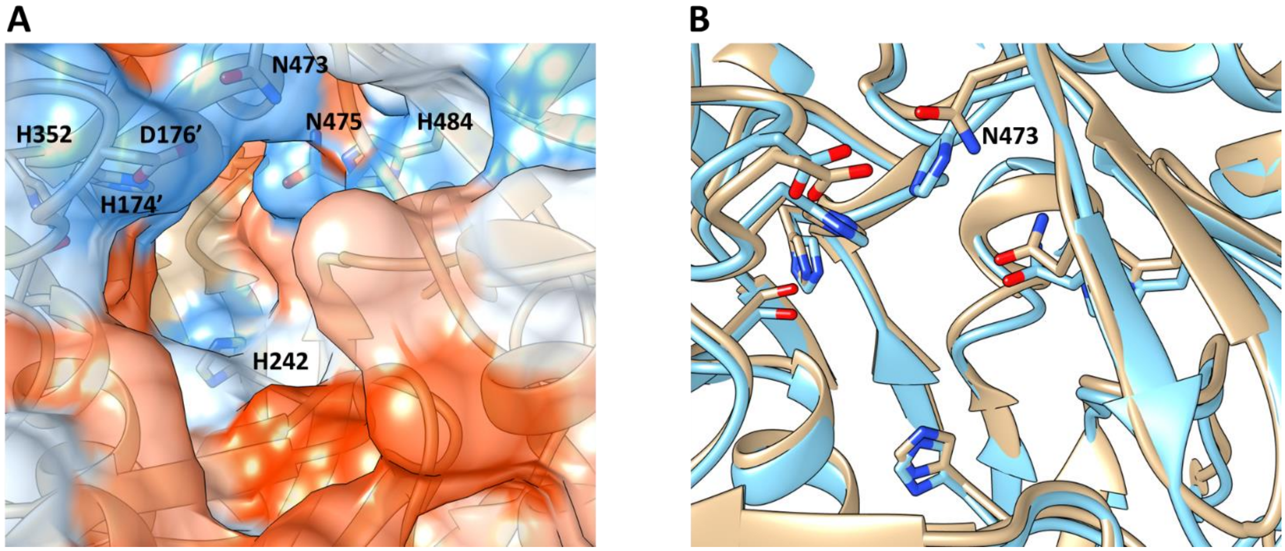

Figure S7. (A) Surface representation of the active site entrance of hSPL colored by hydrophobicity index. Polar and charged residues are colored in blu. (B) Superimposition of AfuSPL (cyan) and hSPL (gold) showing the conservation of polar and charged residues at the active site entrance. All residues are conserved with only one substitution (N473 in hSPL to Histidine in AfuSPL).
